# A Facile Protocol for C(sp^2^)–C(sp^3^) Bond Formation Reactions Toward Functionalized E3 Ligase Ligands

**DOI:** 10.1002/cmdc.202500929

**Published:** 2025-12-16

**Authors:** Anita Maksutova, Thomas M. Geiger, Lorenzo Cianni, Dominika E. Pieńkowska, Jan Gerhartz, Lina Read, Aleša Bricelj, Alexander Herrmann, Maurice Leon Nelles, Yuen Lam Dora Ng, Marcus D. Hartmann, Jan Krönke, Izidor Sosič, Radosław P. Nowak, Michael Gütschow, Christian Steinebach

**Affiliations:** ^1^ Pharmaceutical Institute University of Bonn An der Immenburg 4 DE‐53121 Bonn Germany; ^2^ Institute of Structural Biology University of Bonn Venusberg‐Campus 1 DE‐53127 Bonn Germany; ^3^ Faculty of Pharmacy University of Ljubljana Aškerčeva cesta 7 SI‐1000 Ljubljana Slovenia; ^4^ Max Planck Institute for Biology Max‐Planck‐Ring 1 DE‐72076 Tübingen Germany; ^5^ Interfaculty Institute of Biochemistry University of Tübingen Auf der Morgenstelle 34 DE‐72076 Tübingen Germany; ^6^ Department of Hematology, Oncology and Cancer Immunology Charité—Universitätsmedizin Berlin Hindenburgdamm 30 DE‐12203 Berlin Germany; ^7^ Universitätsmedizin Greifswald Ferdinand‐Sauerbruch‐Straße DE‐17475 Greifswald Germany

**Keywords:** C(sp^2^)–C(sp^3^) bond formation, cereblon, E3 ligands, medicinal chemistry, proteolysis‐targeting chimeras

## Abstract

A straightforward method for creating C(sp^2^)–C(sp^3^) bonds is employed to develop novel cereblon (CRBN) E3 ligase ligands, essential for targeted protein degradation (TPD) applications. While most prior studies focus on biological activities, this work explores how the linker attachment and bond types affect physicochemical stability, binding affinity, and degrading performance. Utilizing *N*‐hydroxyphthalimide (NHP) esters and aryl bromides, a resilient decarboxylative cross‐coupling technique that broadens the available chemical space beyond traditional C(sp^2^)–N connections is developed. Several well‐established and underexplored CRBN binders and their derivatives are synthesized and studied. Binding affinity, aqueous solubility, stability in microsomes, and degradation of typical CRBN ligand off‐targets are then investigated. Selected compounds are transformed into GSPT1‐targeting molecular glue degraders or BRD4‐targeting proteolysis‐targeting chimeras (PROTACs). Benzamide‐based degraders obtained using the new method have a very high ability to break down BRD4. This research shows that C(sp^2^)–C(sp^3^) connections open up new ways to fine‐tune PROTAC characteristics, which unlock degrader chemotypes that were not accessible before. The results demonstrate the importance of synthetic innovation in developing ligands for TPD applications.

## Graphical abstract

A robust C(sp^2^)–C(sp^3^) decarboxylative coupling strategy enables access to new CRBN ligands and degraders with improved physicochemical properties. This synthetic approach is expanding the chemical space beyond C(sp^2^)–N linkages, fine‐tuning proteolysis‐targeting chimera activities and unlocking previously inaccessible degrader chemotypes.

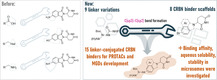

## 1 Introduction

**Figure 1:**
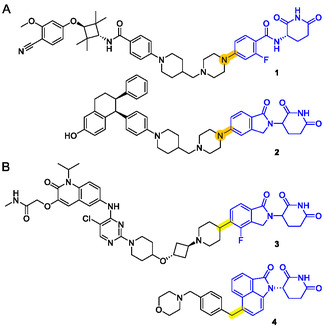
A) Examples of C(sp^2^)–N bonds embedded in successful degraders: (**1**) luxdegalutamide (ARV‐766), an AR‐targeting PROTAC; (**2**) vepdegestrant (ARV‐471), an ER‐targeting PROTAC. B) Examples of C(sp^2^)–C(sp^3^) bonds embedded in successful degraders: (**3**) ARV‐393, a BCL6‐targeting PROTAC; (**4**) cemsidomide (CFT7455), a next‐generation IKZF1/3 MGD.

Targeted protein degradation (TPD) has become one of the most important therapeutic modalities with proteolysis‐targeting chimeras (PROTACs) and molecular glue degraders (MGDs) spearheading the field.^[^ ^1^ ^,^ ^2^ ^]^ In both cases, immunomodulatory

drugs (IMiDs), such as pomalidomide, lenalidomide, and their structural analogs, are the most often utilized building blocks. These molecules play a central role by binding to cereblon (CRBN) and subsequently either gluing neosubstrates or acting as an anchor to bring a desired protein in the proximity of CRBN.^[^ ^1^ ^,^ ^3^ ^]^ In both cases, this results in ubiquitination and degradation of the recruited protein. Recently, the structural diversity of CRBN ligands has increased, and nontraditional binders are gaining traction in the field. The majority of IMiD‐derived and other less conventional CRBN binders are connected to linkers in a chemically limited mode, which constrains the possibilities to accurately modulate binding, stability, solubility, and degradation profiles.^[^ ^4^ ^]^ With a recent shift toward rigid linkers, the C(sp^2^)–N bond connection between the linker and the E3 ligase ligand, typically enabled by the Buchwald–Hartwig protocol,^[^ ^5^ ^,^ ^6^ ^]^ became prevalent (see, for example, **Figure** **1**A). To expand the chemical space and concurrently enable property‐driven optimization, new and straightforward synthetic approaches to alternative linker connection types, such as C(sp^2^)–C(sp^3^) (Figure 1B), could unlock CRBN ligand scaffolds with modified physicochemical properties. In general, motifs rich in sp^3^‐hybridized carbon atoms confer more drug‐like properties.^[^ ^7^ ^]^ To bridge this methodological gap, we developed a robust and functional‐group‐tolerant cross‐coupling method, which was achieved by combining appropriate aryl bromides and *N*‐hydroxyphthalimide (NHP) esters. To venture beyond just making C(sp^2^)–C(sp^3^) bonds, we investigated how this bond type influences a variety of physicochemical and degradation properties of MGDs and selected PROTACs. We are convinced that this work will lead to the expansion of the chemical toolbox for fine‐tuning linker geometry, rigidity, and physicochemical properties of degraders.

## 2 Results and Discussion

**Figure 2:**
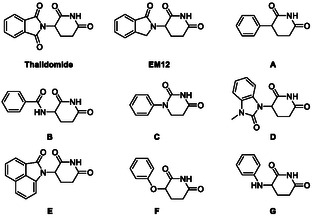
A selection of classical and nontraditional CRBN recruiters investigated in this study.

**Scheme 1:**
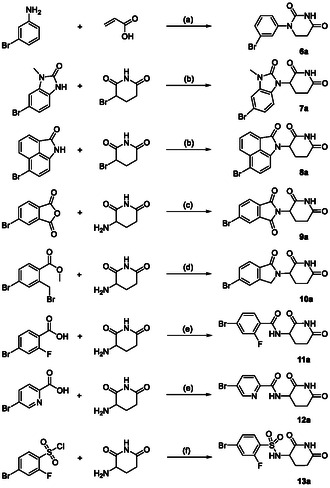
Synthesis of aryl bromides **6a**–**13a**. *Reagents and conditions*: a) (i) acrylic acid, 100 °C, 3 h; (ii) AcOH, 100 °C, 10 min; (iii) urea, 120 °C, 18 h, 46%; b) NaH, DMF/THF, 0 °C to rt or 60 °C, 16 h, 18%–29%; c) NaOAc, AcOH, 120 °C, 4 h, 86%; d) DIPEA, MeCN, 80 °C, 48 h, 67%; e) EDC, HOBt, DMF, rt, 18 h, 74%–77%; f) K_2_CO_3_, THF, 30 °C, 24 h, 17%.

**Scheme 2:**
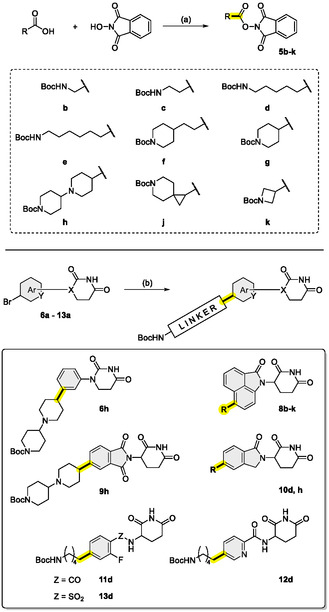
Synthesis of alkyl *N*‐hydroxyphthalimide esters (top) and exemplary application of the C(sp^2^)–C(sp^3^) coupling procedure to cereblon ligands **6**–**13**. *Reagents and conditions*: a) EDC, CH_2_Cl_2_, rt, 16 h, 51%–99%; b) **5**, NiCl_2_(DME), 5‐methoxypicolinimidamide hydrochloride, TBAI, TFA, zinc dust, DMAc, rt, 2 h, 4%–81%.

Although multiple IMiD‐derived and nontraditional CRBN binders for PROTAC development were disclosed recently,^[^ ^6^ ^,^ ^8^, ^9^, ^10^, ^11^ ^]^ we noticed that the majority of reports strongly focused on optimizing biological activity rather than physicochemical properties and stability of the compounds.^[^ ^11^ ^]^ In previous studies, we have identified that the linker attachment point has a significant impact on the stability and neosubstrate degradation features of thalidomide and lenalidomide analogs.^[^ ^4^ ^]^ Accordingly, our present investigation started with assessing the characteristics of the novel undecorated CRBN recruiters **A**–**G** (**Figure** **2**) compared to thalidomide and the related oxoisoindoline EM12. The selected scaffolds appeared frequently in recent PROTAC publications and patents.^[^ ^1^ ^]^ Our investigations comprised in vitro microscale thermophoresis (MST) binding data, cellular CRBN NanoBRET engagement data, biomimetic high‐performance liquid chromatography (HPLC)‐derived parameters (log*D* and human serum albumin (HSA) binding, immobilized artificial membrane chromatography), solubility data, degradation data for the CRBN neosubstrate SALL4, redox reactivity, chemical stability, and microsomal stability results (Table S1, Supporting Information). Our data suggests that the dihydrouracil **C**, the benzo[*d*]imidazole **D**, and the benzo[*cd*]indole derivative **E** are particularly interesting entities for further development. However, as compared to classical IMiDs,^[^ ^3^ ^]^ we noticed a limited availability of synthetic protocols for linker attachment to the aromatic core of such structures. Accordingly, we sought to develop a facile linker attachment procedure for CRBN recruiters such as **A**–**G**. We

hypothesized that these scaffolds are compatible with a nickel‐catalyzed C(sp^2^)–C(sp^3^) bond formation reaction between their respective aryl bromides and alkyl *N*‐hydroxyphthalimide (NHP) esters.^[^ ^12^ ^]^ The decarboxylative cross‐electrophile coupling

reaction has several advantages over previously reported C(sp^2^)–C(sp^3^) bond formation reactions used in the TPD field,^[^ ^8^ ^,^ ^13^ ^,^ ^14^ ^]^ i.e., 1) bromo‐substituted CRBN ligands are readily available for a diverse set of scaffolds (see **Scheme** **1**), 2) NHP esters can be easily generated from *N*‐Boc‐protected carboxylic acids, and 3) the reaction between NHP esters and aryl bromides requires generally versatile reaction setups.

**Figure 3:**
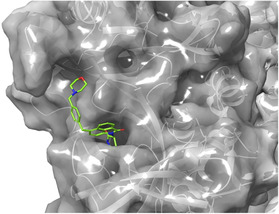
Docked pose for ligand **4** (green) in the thalidomide‐binding pocket of DDB1‐CRBN E3 ubiquitin ligase (PDB 4CI1).

The substituted phenyluracil **6a**, which is considered a privileged CRBN‐binding scaffold due to its nonchiral properties, was readily available from 3‐bromoaniline and acrylic acid.^[^ ^15^ ^]^ Both aryl bromide analogs of **D** and **E** (i.e., **7a** and **8a**) could be obtained in moderate yield via alkylation with 3‐bromopiperidine‐2,6‐dione. To demonstrate the general utility of our novel protocol, we extended the portfolio to classical IMiD scaffolds and synthesized **9a** and **10a** via tandem condensation reactions, as well as **11a**.^[^ ^3^, ^4^, ^5^ ^,^ ^16^ ^]^ Compounds **12a** and **13a** were added to the series due to the promising biological activity of benzamide analogs in which a phenyl‐to‐pyridine substitution or a bioisosteric replacement of the amide moiety was conducted.^[^ ^17^, ^18^, ^19^ ^]^ Following the EDC‐mediated assembly of various NHP ester precursors of type **5** (**Scheme** **2**, top), we subjected aryl bromide‐decorated CRBN ligands **6a**–**13a** to the intended cross‐coupling reaction. The applied nickel‐catalyzed transformation required the presence of catalytic amounts of a picolinimidamide ligand, as well as tetrabutyl‐ammonium iodide (TBAI) and zinc as additives (Scheme 2, bottom).^[^ ^12^ ^]^ However, for all entries except for **7a**, reasonable yields of the desired linker conjugates harboring a terminal Boc group were achieved. Of note, the reaction proceeded well without protecting the glutarimide NH group, and the reaction with an *N*‐alkylated thalidomide analog (data not shown) gave a similar yield. Besides effectively obtaining the desired phenyluracil derivative **6h**, we were able to install a diverse linker library onto the privileged benzo[*cd*]indole scaffold of type **8**. Importantly, rigid moieties containing piperidines (**8g** and **8h**), spirocyclic moieties (**8j**), or azetidine (**8k**) could be conveniently installed at this CRBN‐binding scaffold. For the foremost example (**8g**), a sequential Suzuki coupling with *N*‐Boc‐1,2,3,6‐tetrahydropyridine‐4‐boronic acid, followed by the hydrogenation of the double bond, was typically necessary.^[^ ^20^ ^]^ In **Figure** **3**, compound **4** was docked into the crystal structure of CRBN (PDB: 4CI1), highlighting the proper selection of the linker exit vector. As anticipated, the nickel‐catalyzed C(sp^2^)–C(sp^3^) bond formation reaction is a versatile expansion of the linker chemistries of IMiD derivatives **9** and **10** (Scheme 2). In addition, we could synthesize novel benzamide analogs, such as **11d**, thereby circumventing the C(sp^2^)–N bonds formed after applying Buchwald–Hartwig or Ullmann coupling protocols. Notably, the transformation was also possible in the presence of sulfur atoms or with heteroaromatic moieties, as shown by the successful generation of the benzamide analogous conjugates **12** and **13**.

**Scheme 3:**
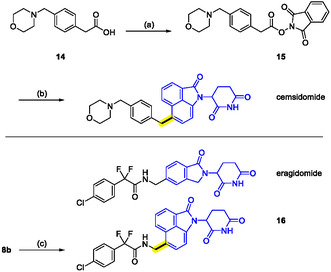
Revisiting the synthesis of (*rac*)‐cemsidomide (top) and application of the C(sp^2^)–C(sp^3^) coupling procedure for discovery of advanced GSPT1 degraders (bottom). *Reagents and conditions*: a) EDC, CH_2_Cl_2_, rt, 16 h, 30%; b) **8a**, NiCl_2_(DME), 5‐methoxypicolinimidamide hydrochloride, TBAI, TFA, zinc dust, DMAc, rt, 2 h, 30%; c) (i) TFA, CH_2_Cl_2_, rt, 2 h; (ii) 2‐(4‐chlorophenyl)−2,2‐difluoroacetic acid, HATU, DIPEA, DMF, rt, 16 h, 45%.

The benzo[*cd*]indoles of type **8** were included in our study due to the outstanding CRBN‐binding data (Table S1, Supporting Information) and the superiority of cemsidomide compared to classical IMiDs such as pomalidomide.^[^ ^21^ ^]^ In the original patent, cemsidomide, which also contains a C(sp^2^)–C(sp^3^) bond, could only be obtained by applying organolithium reagents at ultralow temperatures. In a related work, the benzo[*cd*]indole scaffold was used for the assembly of BRD9‐targeting PROTACs.^[^ ^22^ ^]^ However, the Suzuki‐coupling procedure applied in the work by Duan et al. restricted the exploration of linkers. To demonstrate the utility of our transformations for the assembly of MGDs, we revisited the synthetic route of cemsidomide. Starting from building blocks **8a** and 2‐(4‐(morpholinomethyl)phenyl)acetic acid (**14**), the desired compound could be readily obtained (**Scheme** **3**, top). Encouraged by these results and the known biological activity of cemsidomide, we envisaged replacing the classical IMiD scaffold in eragidomide (CC‐90009), a selective GSPT1 MGD,^[^ ^23^ ^]^ by nontraditional CRBN‐recruiting moieties. The benzyl amine moiety could be easily installed at such scaffolds by employing the NHP ester of *N*‐Boc‐glycine and by utilizing our novel C(sp^2^)–C(sp^3^) coupling procedure (Scheme 3, bottom). Specifically, we converted precursor **8a** to its Boc‐protected linker derivative **8b** (see also Scheme 2, bottom), which was

subsequently deprotected and coupled with 2‐(4‐chlorophenyl)‐2,2‐difluoroacetic acid to give the tailored analog of eragidomide, compound **16**. The two MGDs were profiled for physicochemical properties, cancer cell growth inhibition in Molt4 cells, and GSPT1 degradation (DC_50_, *D*_max_) in Flp293T cells stably expressing GSPT1^389−499^‐GFP fusion proteins (**Table** **1**). As expected, the modification was accompanied by an increase in lipophilicity (clog*D* 2.0 vs. 2.9) and slightly higher plasma protein binding. With respect to the compounds’ biological activity, we observed similar degradation potency of the new analog **16** compared to eragidomide (both exhibiting DC_50_ values of ≈1 nM), but less pronounced maximal degradation plateaus after a 5 h treatment (52% vs. 65% GSPT1^389−499^‐GFP reporter degradation). Surprisingly, compound **16** did not cause pronounced cytotoxicity in Molt4 cells after 5 days of treatment, whereas eragidomide significantly reduced cell viability. This could reflect a higher degradation susceptibility of the GSPT1^389−499^‐GFP degradation reporter than that of the endogenous full‐length GSPT1, leading to an overestimation of degradation activity.

**Table 1:** Overview on physicochemical properties as well as cell growth inhibition and GSPT1 degradation data of MGDs.

Compound	clog*D*_7.4_ a)	%PPBb)	EC_50_ c) (µM)	DC_50_ d) (nM)	*D*_GSPT1_ d) (%)
Eragidomide	2.0	90.0	0.08 ± 0.006	1.5 ± 0.1	65
**16**	2.9	94.5	>10	1.3 ± 0.1	52

a) Calculated distribution coefficient at pH 7.4.

b) HSA binding values were estimated by an HPLC‐based method.

c) Cell growth inhibition data of Molt4 cells treated with MGDs for 5 days. Data represents mean ± SE and *n* = 3.

d) Half‐maximal degradation concentration (DC_50_) and bottom plateau values (*D*_GSPT1_) as determined by measuring GSPT1^389−499^‐GFP fusion protein levels in Flp293T cells after a 5 h treatment of with MGDs. Data represents mean ± SE and *n* = 2.

**Figure 4:**
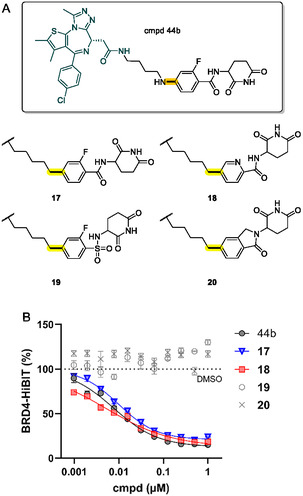
A) Structures of alternative BRD4‐targeting PROTACs obtained via the C(sp^2^)–C(sp^3^) coupling procedure. B) Biological evaluation of PROTACs **17–20** in comparison with benzamide‐type PROTAC 44b from ref. [19].

In previous studies, we explored ortho‐fluoro benzamide‐type CRBN ligands as versatile E3 recruiters with improved physicochemical properties and reduced off‐target effects.^[^ ^19^ ^]^ Therein, we employed a copper‐catalyzed Ullmann reaction protocol to facilitate the C(sp^2^)–N bond formation at the linker exit vector. Out of the previous series of BRD4 PROTACs, compound 44b (**Figure** **4**, top) ranked among the most active representatives.^[^ ^19^ ^]^ However, benzamide‐type ligands had the disadvantage of relatively low CRBN‐binding affinity. Due to the availability of our new protocol for C(sp^2^)–C(sp^3^) bond formations, we aimed to extend the portfolio of BRD4 PROTACs and synthesize a set of highly related compounds. Since the C(sp^2^)–C(sp^3^) coupling procedure in the present work is compatible with aromatic moieties featuring multiple halogen atoms, we were able to synthesize the close analog **17** in which the exit vector nitrogen is replaced by carbon. By analogy, we successfully obtained the pyridine derivative **18** from the heteroaromatic precursor **12d** (see Scheme 2). Considering the clinical advancement of bexobrutideg, a picolinamide‐based BTK degrader, our new synthetic protocol may pave the way for an extended chemical space, including novel linker types.

As a further extension of an interesting report on the bioisosteric replacement of the carbonyl group in lenalidomide‐type compounds,^[^ ^17^ ^]^ we aimed to explore sulfonamide analogs of compound 44b, which was achieved with BRD4 degrader **19**. For a biological head‐to‐head comparison of the PROTAC series, we included compound 44b. Physicochemical property profiles as well as biological activities are provided in **Table** **2**. In contrast to the reference PROTAC, compound **17** displayed higher lipophilicity and HSA binding, which may be attributed to the reduced polarity by replacing the linker junction. Accompanying the alternative linker attachment was a reduction of the hydrogen‐bond donor (HBD) count by one. Of note, the carboxamide HBD in the CRBN‐binding moiety is considered internally satisfied, as we demonstrated previously.^[^ ^19^ ^]^ Accordingly, PROTACs **17** to **19** possess only two unsatisfied HBDs, which is regarded as a cutoff for orally bioavailable PROTACs.^[^ ^24^ ^,^ ^25^ ^]^ The pyridine derivative **18** had a log*D*_7.4_ value of 2.4 and was 92% bound to HSA. Both benzamide derivatives **17** and **18** exhibited excellent degradation potencies (DC_50_ = 10 and 5 nM, respectively). With **18**, the potency of the previously described

**Table 2:** Overview of physicochemical properties, as well as cell growth inhibition and BRD4 degradation data of PROTACs.

Compound	HBDa) count	elog*D*_7.4_ b)	%PPBc)	DC_50_ d) (nM)	*D*_max_ d) (%)	EC_50_ e) (µM)
Cmpd 44b	4 (3*)	2.3	91.8	7.6	87	0.10 ± 0.006
**17**	3 (2*)	2.6	93.3	9.9	79	0.27 ± 0.016
**18**	3 (2*)	2.4	92.0	5.0	88	0.26 ± 0.012
**19**	3 (2*)	2.8	93.9	>1000	<5	n.d.f)
**20**	2	1.8	90.0	>1000	<5	n.d.

a) Number of HBDs. An asterisk indicates the number of unsatisfied HBD.

b) Experimental distribution coefficient at pH 7.4.

c) HSA binding values were estimated by an HPLC‐based method.

d) Half‐maximal degradation concentration (DC_50_) and maximal degradation (*D*_max_) as determined by measuring BRD4‐HiBiT levels after a 5 h treatment of HEK293T BRD4‐HiBiT knock‐in cells with MGDs.

e) Cell growth inhibition data of Molt4 cells treated with PROTACs for 5 days. Data represents mean ± SE and *n* = 3.

f) Not determined.

benzamide‐type PROTAC could even be slightly improved. However, the sulfonamide analog of **17**, i.e., compound **19**, was inferior to its progenitors, which may result from the lower CRBN‐binding capacity of the sulfonamide (see Table S1, Supporting Information). While PROTACs **17**–**19** represent rotationally flexible molecules,^[^ ^19^ ^]^ the isoindolinone derivative **20** constitutes a classical IMiD derivative. Of note, EM‐12‐derived PROTACs harboring a C(sp^2^)–C(sp^3^) linker attachment were previously only accessible via a sequential Sonogashira coupling and reductive saturation of the triple bond.^[^ ^3^ ^]^ The decreased lipophilicity of **20** (Table 2) was accompanied by negligible BRD4 degradation capabilities (DC_50_ > 1000 nM and *D*_max_ < 5%). Finally, we have assessed the antiproliferative activity of all active PROTACs. Molt4 cells were treated with compounds for over 5 days, and viability was evaluated. Cytotoxicity was moderately correlated with BRD4 degradation potencies. Still, the phenotypic correlation may also be influenced by the long‐term effects of BRD4 degradation and the stability of the respective CRBN‐binding scaffolds.^[^ ^26^ ^]^

## 3 Conclusion

Establishing new synthetic protocols for functionalized E3 ligase ligands is crucial for expanding the chemical space of degraders and allowing for property‐driven optimization of lead molecules. Novel CRBN E3 ligase recruiters are on the way, but with an increased complexity of the ligands, alternative yet simple protocols to Suzuki and Buchwald–Hartwig are needed.^[^ ^9^ ^]^ In this study, we first assessed a diverse set of undecorated CRBN ligands for their suitability as PROTAC precursors. We focused on their physicochemical profiles, stability data, and CRBN‐binding affinity. For the most promising candidates, we have then revisited synthetic protocols toward bromo‐substituted derivatives thereof. In contrast to previous work employing Ni‐catalyzed cross‐coupling reactions, our study has been concentrated on the opportunity for linker attachments to E3 ligase handles.^[^ ^27^ ^]^ Starting from commercially available alkanoic acids, we next synthesized a vast library of NHP esters, versatile linker building blocks. The applied protocol for a decarboxylative cross‐electrophile coupling reaction, which facilitates an unprecedented C(sp^2^)–C(sp^3^) bond formation in CRBN recruiters, allowed us to assemble novel PROTAC and MGD building blocks. In the case of **16**, we were able to synthesize a naphtholactam derivative of eragidomide with outstanding GSPT1 degradation activity. For the synthesis of BRD4‐targeting PROTACs, we could extend the chemical space for benzamide‐type PROTACs. Importantly, by switching from an Ullmann‐type coupling reaction toward 44b, our C(sp^2^)–C(sp^3^) bond formation reaction yielded close carbon counterparts, thereby further eliminating one H‐bond donor in **17**. The protocol is applicable to a wide range of aromatic substrates, including heterocyclic scaffolds, such as the pyridine derivative **18**, or sulfur‐containing compounds such as **19**. The protocol requires less strict handling under inert conditions and is not dependent on electrochemical or photochemical reaction setups.^[^ ^14^ ^,^ ^28^ ^]^ Therefore, we believe that this study will aid CRBN‐based degrader design by refocusing on the most promising E3 recruiters and by unlocking novel synthetic strategies for property‐driven PROTAC discovery campaigns.

## 4 Experimental Section

4.1

4.1.1

##### Chemical Synthesis: General Remarks

Preparative column chromatography was performed using Merck silica gel 60 (0.063–0.200 mm) or using an automated flash chromatography (FC) system puriFlash XS 520 Plus. Melting points were determined on a Büchi 510 oil bath apparatus or on a Reichelt hot‐stage apparatus and were uncorrected. ^1^H NMR and ^13^C NMR spectra were recorded on a Bruker Avance 400 MHz NMR spectrometer, Bruker Avance 500 MHz NMR spectrometer, or a Bruker Avance III 600 MHz NMR spectrometer, respectively. NMR spectra were processed and analyzed in MestReNova. Chemical shifts are given in parts per million (ppm), coupling constants *J* are given in hertz, and spin multiplicities are given as s (singlet), d (doublet), t (triplet), q (quartet), or m (multiplet). In case of overlapping extraneous solvent peaks, multiplet analyses in ^1^H NMR spectra were performed using quantitative Global Spectral Deconvolution (qGSD). Resonance assignments were made based on one‐ and two‐dimensional NMR techniques, which include ^1^H, ^13^C, DEPT, HSQC, and HMBC experiments. The purity and identity of the compounds were determined by HPLC‐UV obtained on an LC‐MS instrument (Agilent Infinity Lab LC/MSD‐system with ESI‐source coupled with an Agilent HPLC 1260 Infinity II). For LC/MS analysis, an EC50/2 Nucleodur C18 Gravity 3 μm column (Macherey‐Nagel) was used. The column temperature was 40 °C. HPLC conditions started with 90% H_2_O containing 2 mM NH_4_Ac. The gradient ramped up to 100% MeCN in 10 min, followed by further flushing with 100% MeCN for 5 min. The flow rate was 0.5 mL min^–1^. The samples were dissolved in H_2_O, MeOH, or MeCN (≈1 mg mL^–1^), and 2 μL sample solution was injected. Positive total ion scans were observed from 100 to 1000 m z^–1^ (or more if necessary), and UV absorption was detected from 190 to 600 nm using a diode array detector (DAD). The purity was determined at 220–600 nm, unless indicated otherwise. All compounds that were evaluated in biological assays were ≥95% pure by LC/MS analysis.

##### rac‐Cemsidomide (4)

This compound was prepared using the General Procedure II (0.30 mmol scale), NHP‐ester **15** (0.11 g), and aryl bromide **11a** (0.14 g). After evaporation of the solvent, the crude product was purified with FC (gradient from 0 to 6% MeOH in CH_2_Cl_2_) to get the desired compound as a yellow solid. Yield: 0.04 g (30%); m.p. 231–233 °C; *R*_f_ = 0.21 (5% MeOH in CH_2_Cl_2_); **LC‐MS** (ESI) (90% H_2_O to 100% MeCN in 10 min, then 100% MeCN to 15 min, DAD 220–600 nm), *t*_R_ = 5.89 min, 98% purity, *m*/*z* [M + H]^+^ calcd for C_28_H_27_N_3_O_4_, 470.21; found, 470.0.

##### General Procedure I for the Synthesis of NHP‐Ester Derivatives

To a stirred solution of an *N*‐protected amino acid (1.0 eq), *N*‐hydroxyphthalimide (1.1 eq), and 4‐dimethylaminopyridine (0.1 eq) in CH_2_Cl_2_ (10 mL mmol^–1^), a suspension of 1‐ethyl‐3‐(3‐dimethylaminopropyl)carbodiimide (1.2 eq) in CH_2_Cl_2_ (3 mL mmol^–1^) was added dropwise. The resulting solution was allowed to stir overnight at rt. The reaction mixture was washed with HCl solution (1 mol L^−1^, 5 mL mmol^–1^) and water (3 × 10 mL). The organic layer was dried over Na_2_SO_4_, filtered, and evaporated. If necessary, purification was performed using FC and mixtures of EtOAc/cyclohexane as eluents.

##### 1,3‐Dioxoisoindolin‐2‐yl (tert‐Butoxycarbonyl)glycinate (5b)

This compound was prepared using the General Procedure I (1.5 mmol scale) and Boc‐Gly‐OH (0.26 g). After evaporation of the solvent, a colorless solid was obtained that was used without further purification. Yield: 0.24 g (51%); m.p. 190–194 °C; *R*_f_ = 0.20 (80% EtOAc in petroleum ether); **LC‐MS** (ESI) (90% H_2_O to 100% MeCN in 10 min, then 100% MeCN to 15 min, DAD 220–600 nm), *t*_R_ = 6.30 min, 96% purity, *m*/*z* [M + H − Boc]^+^ calcd for C_10_H_8_N_2_O_4_, 221.06; found, 221.1.

##### 1,3‐Dioxoisoindolin‐2‐yl 3‐((tert‐Butoxycarbonyl)amino)propanoate (5c)

This compound was prepared using the General Procedure I (1.5 mmol scale) and 3‐((*tert*‐butoxycarbonyl)amino)propanoic acid (0.28 g). After evaporation of the solvent, a colorless solid was obtained that was used without further purification. Yield: 0.33 g (99%); m.p. 107–110 °C; *R*_f_ = 0.35 (30% EtOAc in petroleum ether); **LC‐MS** (ESI) (90% H_2_O to 100% MeCN in 10 min, then 100% MeCN to 15 min, DAD 220–600 nm), *t*_R_ = 6.51 min, 91% purity, *m*/*z* [M + H − Boc]^+^ calcd for C_11_H_10_N_2_O_4_, 235.07; found, 235.5.

##### 1,3‐Dioxoisoindolin‐2‐yl 6‐((tert‐Butoxycarbonyl)amino)hexanoate (5d)

This compound was prepared using the General Procedure I (1.5 mmol scale) and 6‐((*tert*‐butoxycarbonyl)amino)hexanoic acid (0.35 g). After evaporation of the solvent, a colorless solid was obtained that was used without further purification. Yield: 1.03 g (92%); m.p. 83–86 °C; *R*_f_ = 0.35 (30% EtOAc in petroleum ether); **LC‐MS** (ESI) (90% H_2_O to 100% MeCN in 10 min, then 100% MeCN to 15 min, DAD 220–600 nm), *t*_R_ = 7.31 min, 91% purity, *m*/*z* [M + H − Boc]^+^ calcd for C_14_H_16_N_2_O_4_, 277.12; found, 277.5.

##### 1,3‐Dioxoisoindolin‐2‐yl 7‐((tert‐Butoxycarbonyl)amino)heptanoate (5e)

This compound was prepared using the General Procedure I (1.5 mmol scale) and 7‐((*tert*‐butoxycarbonyl)amino)heptanoic acid (0.37 g). After evaporation of the solvent, a colorless solid was obtained that was used without further purification. Yield: 0.39 g (67%); m.p. 71–73 °C; *R*_f_ = 0.38 (30% EtOAc in petroleum ether); **LC‐MS** (ESI) (90% H_2_O to 100% MeCN in 10 min, then 100% MeCN to 15 min, DAD 220–600 nm), *t*_R_ = 7.34 min, 92% purity, *m*/*z* [M + H − Boc]^+^ calcd for C_15_H_18_N_2_O_4_, 291.13; found, 291.2; **HRMS** (ESI) *m*/*z* [M + H]^+^ calcd for C_20_H_27_N_2_O_6_, 391.1864; found, 391.1846.

##### Tert‐Butyl 4‐(3‐((1,3‐Dioxoisoindolin‐2‐yl)oxy)‐3‐Oxopropyl)piperidine‐1‐Carboxylate (5f)

This compound was prepared using the General Procedure I (1.5 mmol scale) and 3‐(1‐(*tert*‐butoxycarbonyl)piperidin‐4‐yl)propanoic acid (0.39 g). After evaporation of the solvent, a colorless solid was obtained that was used without further purification. Yield: 0.42 g (70%); m.p. 116–118 °C; *R*_f_ = 0.38 (30% EtOAc in petroleum ether); **LC‐MS** (ESI) (90% H_2_O to 100% MeCN in 10 min, then 100% MeCN to 15 min, DAD 220–600 nm), *t*_R_ = 8.09 min, 91% purity, *m*/*z* [M + H]^+^ calcd for C_21_H_27_N_2_O_6_, 403.19; found, 403.2.

##### 1‐(Tert‐Butyl) 4‐(1,3‐Dioxoisoindolin‐2‐yl)piperidine‐1,4‐Dicarboxylate (5g)

This compound was obtained from commercial sources (BLD Pharmatech GmbH, Reinbek, Germany). Colorless solid; m.p. 106–108 °C; *R*_f_ = 0.43 (30% EtOAc in petroleum ether); **LC‐MS** (ESI) (90% H_2_O to 100% MeCN in 10 min, then 100% MeCN to 15 min, DAD 220–600 nm), *t*_R_ = 7.56 min, 98% purity, *m*/*z* [M + H]^+^ calcd for C_19_H_22_N_2_O_6_, 319.09; found, 319.2.

##### 1′‐(Tert‐Butyl) 4‐(1,3‐Dioxoisoindolin‐2‐l) [1,4′‐Bipiperidine]‐1′,4‐Dicarboxylate (5h)

This compound was prepared using the General Procedure I (1.5 mmol scale) and 1^′^‐(*tert*‐butoxycarbonyl)‐[1,4^′^‐bipiperidine]‐4‐carboxylic acid (0.47 g). After evaporation of the solvent, a yellow solid was obtained that was used without further purification. Yield: 0.59 g (86%); m.p. 180–182 °C (dec.); *R*_f_ = 0.38 (30% EtOAc in petroleum ether); **LC‐MS** (ESI) (90% H_2_O to 100% MeCN in 10 min, then 100% MeCN to 15 min, DAD 220–600 nm), *t*_R_ = 7.13 min, 79% purity, *m*/*z* [M + H]^+^ calcd for C_24_H_32_N_3_O_6_, 458.23; found, 458.4.

##### rac‐6‐(Tert‐Butyl) 1‐(1,3‐Dioxoisoindolin‐2‐yl) 6‐Azaspiro[2.5]octane‐1,6‐Dicarboxylate (5j)

This compound was prepared using the General Procedure I (1.5 mmol scale) and 6‐(*tert*‐butoxycarbonyl)‐6‐azaspiro[2.5]octane‐1‐carboxylic acid (0.38 g). After evaporation of the solvent, a colorless solid was obtained that was used without further purification. Yield: 0.40 g (99%); m.p. 137–140 °C; *R*_f_ = 0.39 (30% EtOAc in petroleum ether); **LC‐MS** (ESI) (90% H_2_O to 100% MeCN in 10 min, then 100% MeCN to 15 min, DAD 220–600 nm), *t*_R_ = 7.97 min, 83% purity, *m*/*z* [M + H − Boc]^+^ calcd for C_16_H_16_N_2_O_4_, 301.12; found, 301.1.

##### 1‐(Tert‐Butyl) 3‐(1,3‐Dioxoisoindolin‐2‐yl) Azetidine‐1,3‐Dicarboxylate (5k)

This compound was prepared using the General Procedure I (1.5 mmol scale) and 6‐(*tert*‐butoxycarbonyl)‐6‐azaspiro[2.5]octane‐1‐carboxylic acid (0.38 g). After evaporation of the solvent, a white solid was obtained that was used without further purification. Yield: 0.46 g (88%); m.p. 156–158 °C; **LC‐MS** (ESI) (90% H_2_O to 100% MeCN in 10 min, then 100% MeCN to 15 min, DAD 220–600 nm), *t*_R_ = 6.94 min, 76% purity, *m*/*z* [M + H]^+^ calcd for C_12_H_10_N_2_O_4_, 247.07; found, 247.1.

##### 1‐(3‐Bromophenyl)dihydropyrimidine‐2,4(1H,3H)‐Dione (6a)

3‐Bromoaniline (2 mmol) was suspended in acrylic acid (9.8 mmol), and the mixture was heated to 100 °C for 3 h. The reaction mixture was cooled to rt, AcOH (2 mL) was added, and the mixture was heated to 100 °C for 10 min, before urea (12 mmol) was added. The reaction mixture was stirred at 120 °C for 18 h. After cooling to room temperature, the reaction mixture was poured onto ice‐cold 1 M HCl aq. solution (10 mL) and stirred in an ice bath for 3 h. The resulting suspension was filtered and dried to get **6a** as a colorless solid. Yield: 0.25 g (46%); m.p. 175–177 °C; *R*_f_ = 0.28 (60% EtOAc in petroleum ether); **LC‐MS** (ESI) (90% H_2_O to 100% MeCN in 10 min, then 100% MeCN to 15 min, DAD 220–600 nm), *t*_R_ = 4.22 min, 97% purity, *m*/*z* [M + H]^+^ calcd for C_10_H_10_^79^BrN_2_O_2_, 268.99; found, 269.0.

##### General Procedure II for the Cross‐Electrophile Coupling^[^ ^12^ ^]^

A mixture of the respective aryl bromide (1.0 eq), the *N*‐hydroxyphthalimide ester (1.1 eq), zinc dust (10 eq), nickel(II) chloride·glyme (0.20 eq), 5‐methoxypicolinimidamide hydrochloride (0.20 eq), TBAI (1.0 eq), and a stirring bar was sealed in a vial outfitted with a pierceable septum. The vial was evacuated and backfilled with argon three times. The mixture was then treated with DMAc followed by a freshly prepared 10% solution of TFA in DMAc (0.50 eq of TFA) and vigorously stirred (>1100 rpm) at rt under argon for 2 h (total DMAc volume: 2 mL mmol^–1^). The reaction mixture was then diluted with ethyl acetate (2 mL mmol^–1^) and briefly shaken. The mixture was filtered through a short plug of celite, eluting with ethyl acetate (100 mL). The filtrate was washed with EDTA disodium salt/NaHCO_3_ solution (2 × 10 mL mmol^–1^), 0.1 N NaOH (3 × 10 mL  mmol^–1^), and brine (10 mL mmol^–^^1^). The organic layer was dried over Na_2_SO_4_, filtered, and evaporated.

##### Tert‐Butyl 4‐(3‐(2,4‐Dioxotetrahydropyrimidin‐1(2H)‐yl)phenyl)‐[1,4^′^‐Bipiperidine]‐1^′^‐Carboxylate (6h)

This compound was prepared using the General Procedure II (0.50 mmol scale), NHP‐ester **5h** (0.25 g), and aryl bromide **6a** (0.13 g). After evaporation of the solvent, the crude product was purified with FC (gradient from CH_2_Cl_2_ to 50% MeOH in CH_2_Cl_2_) to get the desired compound as a colorless oil. Yield: 60 mg (38%); *R*_f_ = 0.45 (10% MeOH in CH_2_Cl_2_); **LC‐MS** (ESI) (90% H_2_O to 100% MeCN in 10 min, then 100% MeCN to 15 min, DAD 220–600 nm), *t*_R_ = 5.01 min, 97% purity, *m*/*z* [M + H]^+^ calcd for C_25_H_37_N_4_O_4_, 457.28; found, 457.3.

##### 3‐(5‐Bromo‐3‐Methyl‐2‐Oxo‐2,3‐Dihydro‐1H‐Benzo[d]imidazol‐1‐yl)piperidine‐2,6‐Dione (7a)

6‐Bromo‐1‐methyl‐1,3‐dihydro‐2*H*‐benzo[*d*]imidazol‐2‐one (0.91 g, 4.0 mmol) was dissolved in dry DMF (20 mL), and NaH (60% dispersion in mineral oil, 0.19 g, 4.8 mmol) was added portionwise at 0 °C under a nitrogen atmosphere. After 15 min,3‐bromopiperidine‐2,6‐dione (0.42 g, 2.0 mmol) in dry DMF (2 mL) was added dropwise at 0 °C. Subsequently, the reaction mixture was stirred at rt for 16 h. It was quenched with ice water (50 mL), extracted with EtOAc (3 × 50 mL), washed with brine (50 mL), and dried over Na_2_SO_4_. After evaporation of the solvent, the crude product was purified with FC (gradient from 50% EtOAc in cyclohexane to EtOAc) to get the desired compound as a gray solid. Yield: 0.12 g (18%); m.p. >250 °C; *R*_f_ = 0.26 (EtOAc); **LC‐MS** (ESI) (90% H_2_O to 100% MeCN in 10 min, then 100% MeCN to 15 min, DAD 220–600 nm), *t*_R_ = 4.72 min, 99% purity, *m*/*z* [M + H]^+^ calcd for C_13_H_13_^79^BrN_3_O_3_, 338.01; found, 338.0.

##### 3‐(6‐Bromo‐2‐Oxobenzo[cd]indol‐1(2H)‐yl)piperidine‐2,6‐Dione (8a)

To a solution of NaH (60% dispersion in mineral oil, 0.40 g, 10 mmol) in DMF (15 mL) and THF (100 mL) was first added 6‐bromobenzo[*cd*]indol‐2(1*H*)‐one (2.48 g, 10 mmol, 1.0 eq), and after 15 min 3‐bromopiperidine‐2,6‐dione (2.30 g, 12  mmol) at 0 °C. After 20 min, the reaction mixture was heated to 60 °C for 2 h. After cooling, it was quenched with ice water (100 mL), extracted with EtOAc (3 × 100 mL), washed with brine (100 mL), and dried over Na_2_SO_4_. After evaporation of the solvent, the crude product was purified with FC (gradient from CH_2_Cl_2_ to 20% CH_3_CN in CH_2_Cl_2_) to get the desired compound as a yellow solid. Yield: 1.04 g (29%); m.p. 228 °C (decomp.); *R*_f_ = 0.43 (60% EtOAc in cyclohexane); **LC‐MS** (ESI) (90% H_2_O to 100% MeCN in 10 min, then 100% MeCN to 15 min, DAD 220–600 nm), *t*_R_ = 5.75 min, 97% purity, *m*/*z* [M + H]^+^ calcd for C_16_H_12_^79^BrN_2_O, 359.00; found, 358.9.

##### Tert‐Butyl ((1‐(2,6‐Dioxopiperidin‐3‐yl)‐2‐Oxo‐1,2‐Dihydro‐Benzo[cd]indol‐6‐l)methyl)carbamate (8b)

This compound was prepared using the General Procedure II (0.50 mmol scale), NHP‐ester **5b** (0.18 g), and aryl bromide **8a** (0.18 g). After evaporation of the solvent, the crude product was purified with FC (gradient from 0 to 50% EtOAc in cyclohexane) to get the desired compound as a yellow solid. Yield: 0.08 g (40%); m.p. 149–152 °C; *R*_f_ = 0.44 (80% EtOAc in petroleum ether); **LC‐MS** (ESI) (90% H_2_O to 100% MeCN in 10 min, then 100% MeCN to 15 min, DAD 220–600 nm), *t*_R_ = 5.73 min, 99% purity, *m*/*z* [M + H]^+^ calcd for C_22_H_24_N_3_O_5_, 410.17; found, 410.5; **HRMS** (ESI) *m*/*z* [M + H]^+^ calcd for C_22_H_24_N_3_O_5_, 410.1710; found, 410.1702.

##### Tert‐Butyl (2‐(1‐(2,6‐Dioxopiperidin‐3‐l)‐2‐Oxo‐1,2‐Dihydro‐Benzo[cd]indol‐6‐l)ethyl)carbamate (8c)

This compound was prepared using the General Procedure II (0.50 mmol scale), NHP‐ester **5c** (0.18 g), and aryl bromide **8a** (0.18 g). After evaporation of the solvent, the crude product was purified with FC (gradient from 0 to 50% EtOAc in cyclohexane) to get the desired compound as a yellow solid. Yield: 0.12 g (56%); *R*_f_ = 0.13 (60% EtOAc in petroleum ether); **LC‐MS** (ESI) (90% H_2_O to 100% MeCN in 10 min, then 100% MeCN to 15 min, DAD 220–600 nm), *t*_R_ = 5.81 min, 99% purity, *m*/*z* [M + H − Boc]^+^ calcd for C_18_H_17_N_3_O_3_, 324.13; found, 324.2; **HRMS** (ESI) *m*/*z* [M + H + NH_4_]^+^ calcd for C_23_H_26_N_3_O_5_, 441.2132; found, 441.2097.

##### Tert‐Butyl (5‐(1‐(2,6‐Dioxopiperidin‐3‐yl)‐2‐Oxo‐1,2‐Dihydro‐Benzo[cd]indol‐6‐l)pentyl)carbamate (8d)

This compound was prepared using the General Procedure II (0.30 mmol scale), NHP‐ester **5d** (0.12 g), and aryl bromide **8a** (0.11 g). After evaporation of the solvent, the crude product was purified with FC (gradient from 0 to 2% MeOH in CH_2_Cl_2_) to get the desired compound as a yellow solid. Yield: 90 mg (61%); *R*_f_ = 0.14 (2% MeOH in CH_2_Cl_2_); **LC‐MS** (ESI) (90% H_2_O to 100% MeCN in 10 min, then 100% MeCN to 15 min, DAD 220–600 nm), *t*_R_ = 6.88 min, 99% purity, *m*/*z* [M + H − Boc]^+^ calcd for C_21_H_23_N_3_O_3_, 366.18; found, 366.3.

##### Tert‐Butyl (6‐(1‐(2,6‐Dioxopiperidin‐3‐yl)‐2‐Oxo‐1,2‐Dihydro‐Benzo[cd]indol‐6‐yl)hexyl)carbamate (8e)

This compound was prepared using the General Procedure II (0.50 mmol scale), NHP‐ester **5e** (0.21 g), and aryl bromide **8a** (0.18 g). After evaporation of the solvent, the crude product was purified with FC (gradient from 0 to 50% EtOAc in cyclohexane) to get the desired compound as a yellow solid. Yield: 0.13 g (52%); *R*_f_ = 0.26 (60% EtOAc in petroleum ether); **LC‐MS** (ESI) (90% H_2_O to 100% MeCN in 10 min, then 100% MeCN to 15 min, DAD 220–600 nm), *t*_R_ = 7.24 min, 99% purity, *m*/*z* [M + H − Boc]^+^ calcd for C_22_H_25_N_3_O_3_, 380.20; found, 380.6.

##### Tert‐Butyl 4‐(2‐(1‐(2,6‐Dioxopiperidin‐3‐yl)‐2‐Oxo‐1,2‐Dihydro‐Benzo[cd]indol‐6‐yl)ethyl)piperidine‐1‐Carboxylate (8f)

This compound was prepared using the General Procedure II (0.50 mmol scale), NHP‐ester **5f** (0.22 g) and aryl bromide **8a** (0.18 g). After evaporation of the solvent, the crude product was purified with FC (gradient from 0 to 50% EtOAc in cyclohexane) to get the desired compound as a yellow solid. Yield: 0.13 g (53%); m.p. 90–93 °C; *R*_f_ = 0.33 (60% EtOAc in petroleum ether); **LC‐MS** (ESI) (90% H_2_O to 100% MeCN in 10 min, then 100% MeCN to 15 min, DAD 220–600 nm), *t*_R_ = 7.54 min, 98% purity, *m*/*z* [M + H − Boc]^+^ calcd for C_23_H_25_N_3_O_3_, 392.20; found, 392.8; **HRMS** (ESI) *m*/*z* [M + H]^+^ calcd for C_28_H_34_N_3_O_5_, 492.2493; found, 492.2488.

##### Tert‐Butyl 4‐(1‐(2,6‐Dioxopiperidin‐3‐yl)‐2‐Oxo‐1,2‐Dihydro‐Benzo[cd]indol‐6‐yl)piperidine‐1‐Carboxylate (8g)

This compound was prepared using the General Procedure II (0.50 mmol scale), NHP‐ester **5g** (0.17 g), and aryl bromide **8a** (0.18 g). After evaporation of the solvent, the crude product was purified with FC (gradient from 0% to 50% EtOAc in cyclohexane) to get the desired compound as a yellow solid. Yield: 90 mg (41%); *R*_f_ = 0.49 (80% EtOAc in petroleum ether); **LC‐MS** (ESI) (90% H_2_O to 100% MeCN in 10 min, then 100% MeCN to 15 min, DAD 220–600 nm), *t*_R_ = 6.84 min, 94% purity, *m*/*z* [M + H − Boc]^+^ calcd for C_21_H_21_N_3_O_3_, 364.17; found, 364.6; **HRMS** (ESI) *m*/*z* [M + H]^+^ calcd for C_26_H_30_N_3_O_5_, 464.2180; found, 464.2169.

##### Tert‐Butyl 4‐(1‐(2,6‐Dioxopiperidin‐3‐yl)‐2‐Oxo‐1,2‐Dihydro‐Benzo[cd]indol‐6‐yl)‐[1,4’‐Bipiperidine]‐1’‐Carboxylate (8h)

This compound was prepared using the General Procedure II (0.40 mmol scale), NHP‐ester **5h** (0.20 g), and aryl bromide **8a** (0.14 g). After evaporation of the solvent, the crude product was purified with FC (gradient from 0 to 10% MeOH in CH_2_Cl_2_) to get the desired compound as a yellow solid. Yield: 37 mg (17%); m.p. 236 °C (decomp.); *R*_f_ = 0.27 (10% MeOH in CH_2_Cl_2_); **LC‐MS** (ESI) (90% H_2_O to 100% MeCN in 10 min, then 100% MeCN to 15 min, DAD 220–600 nm), *t*_R_ = 6.08 min, 98% purity, *m*/*z* [M + H]^+^ calcd for C_31_H_39_N_4_O_5_, 547.29; found, 547.3; **HRMS** (ESI) *m*/*z* [M + H]^+^ calcd for C_31_H_39_N_4_O_5_, 547.2915; found, 547.2936.

##### rac‐Tert‐Butyl 1‐(1‐(2,6‐Dioxopiperidin‐3‐yl)‐2‐Oxo‐1,2‐Dihydro‐Benzo[cd]indol‐6‐yl)‐6‐Aza‐Spiro[2.5]octane‐6‐Carboxylate (8j)

This compound was prepared using the General Procedure II (0.50 mmol scale), NHP‐ester **5j** (0.22 g), and aryl bromide **8a** (0.18 g). After evaporation of the solvent, the crude product was purified with FC (gradient from 0 to 40% EtOAc in cyclohexane) to get the desired compound as a yellow solid. Yield: 10 mg (4%); *R*_f_ = 0.11 (2% MeOH in CH_2_Cl_2_); **LC‐MS** (ESI) (90% H_2_O to 100% MeCN in 10 min, then 100% MeCN to 15 min, DAD 220–600 nm), *t*_R_ = 7.61 min, 94% purity, *m*/*z* [M + H − Boc]^+^ calcd for C_23_H_23_N_3_O_3_, 390.1812; found, 390.6; **HRMS** (ESI) *m*/*z* [M + H]^+^ calcd for C_28_H_32_N_3_O_5_, 490.2336; found, 490.2325.

##### Tert‐Butyl 3‐(1‐(2,6‐Dioxopiperidin‐3‐yl)‐2‐Oxo‐1,2‐Dihydro‐Benzo[cd]indol‐6‐yl)azetidine‐1‐Carboxylate (8k)

This compound was prepared using the General Procedure II (0.50 mmol scale), NHP‐ester **5k** (0.19 g), and aryl bromide **8a** (0.18 g). After evaporation of the solvent, the crude product was purified with FC (gradient from 0% to 60% EtOAc in cyclohexane) to get the desired compound as a yellow solid. Yield: 0.04 g (20%); *R*_f_ = 0.48 (80% EtOAc in petroleum ether); **LC‐MS** (ESI) (90% H_2_O to 100% MeCN in 10 min, then 100% MeCN to 15 min, DAD 220–600 nm), *t*_R_ = 6.20 min, 95% purity, *m*/*z* [M + H]^+^ calcd for C_24_H_26_N_3_O_5_, 436.19; found, 436.6; **HRMS** (ESI) *m*/*z* [M + H]^+^ calcd for C_24_H_26_N_3_O_5_, 436.1867; found, 436.1821.

##### 5‐Bromo‐2‐(2,6‐Dioxopiperidin‐3‐yl)isoindoline‐1,3‐Dione (9a)

This compound was prepared as we reported previously.^[^ ^4^ ^]^

##### Tert‐Butyl 4‐(2‐(2,6‐Dioxopiperidin‐3‐yl)‐1,3‐Dioxoisoindolin‐5‐yl)‐[1,4^′^‐Bipiperidine]‐1^′^‐Carboxylate (9h)

This compound was prepared using the General Procedure II (0.50 mmol scale), NHP‐ester **5h** (0.25 g), and aryl bromide **9a** (0.17 g). After evaporation of the solvent, the crude product was purified with FC (gradient from 0% to 10% MeOH in CH_2_Cl_2_) to get the desired compound as a yellow solid. Yield: 0.12 g (47%); m.p. 147–149 °C; *R*_f_ = 0.44 (10% MeOH in CH_2_Cl_2_); **LC‐MS** (ESI) (90% H_2_O to 100% MeCN in 10 min, then 100% MeCN to 15 min, DAD 220–600 nm), *t*_R_ = 5.80 min, 98% purity, *m*/*z* [M + H]^+^ calcd for C_28_H_37_N_4_O_6_, 525.27; found, 525.3; **HRMS** (ESI) *m*/*z* [M + H]^+^ calcd for C_28_H_37_N_4_O_6_, 525.2708; found, 525.2733.

##### 3‐(5‐Bromo‐1‐Oxoisoindolin‐2‐yl)piperidine‐2,6‐Dione (10a)

This compound was prepared as reported previously.^[^ ^5^ ^]^ In brief, DIPEA (1.16 g, 1.57 mL, 9.0 mmol) was added to methyl 4‐bromo‐2‐(bromomethyl)benzoate (0.92 g, 3.0 mmol) and 3‐amino‐piperidine‐2,6‐dione hydrochloride (0.74 g, 4.5 mmol) in MeCN (20 mL) under a nitrogen atmosphere. The resulting suspension was stirred at 80 °C for 48 h. The reaction mixture was cooled to rt and filtered. The crude product was purified with FC (gradient from 50% EtOAc in cyclohexane to EtOAc) to get the desired compound as a blue solid. Yield: 0.65 g (67%); m.p. >250 °C; *R*_f_ = 0.26 (60% EtOAc in petroleum ether); **LC‐MS** (ESI) (90% H_2_O to 100% MeCN in 10 min, then 100% MeCN to 15 min, DAD 220–600 nm), *t*_R_ = 4.31 min, 99% purity, *m*/*z* [M + H]^+^ calcd for C_13_H_12_BrN_2_O_3_, 323.00; found, 323.0.

##### Tert‐Butyl (5‐(2‐(2,6‐Dioxopiperidin‐3‐yl)‐1‐Oxoisoindolin‐5‐yl)pentyl)carbamate (10d)

This compound was prepared using the General Procedure II (0.50 mmol scale), NHP‐ester **5d** (0.21 g), and aryl bromide **10a** (0.16 g). After evaporation of the solvent, the crude product was purified with FC (gradient from 0% to 5% MeOH in CH_2_Cl_2_) to get the desired compound as a white solid. Yield: 0.11 g (36%); m.p. 182–184 °C; *R*_f_ = 0.61 (10% MeOH in CH_2_Cl_2_); **LC‐MS** (ESI) (90% H_2_O to 100% MeCN in 10 min, then 100% MeCN to 15 min, DAD 220–600 nm), *t*_R_ = 5.99 min, 99% purity, *m*/*z* [M − H]^−^ calcd for C_23_H_32_N_3_O_5_, 428.22; found, 428.2; **HRMS** (ESI) *m*/*z* [M + H]^+^ calcd for C_23_H_32_N_3_O_5_, 430.2336; found, 430.2336.

##### Tert‐Butyl 4‐(2‐(2,6‐Dioxopiperidin‐3‐yl)‐1‐Oxoisoindolin‐5‐yl)‐[1,4^′^‐Bipiperidine]‐1^′^‐Carboxylate (10h)

This compound was prepared using the General Procedure II (0.50 mmol scale), NHP‐ester **5h** (0.25 g), and aryl bromide **10a** (0.16 g). After evaporation of the solvent, the crude product was purified with FC (gradient from 0% to 50% MeOH in CH_2_Cl_2_) to get the desired compound as an orange solid. Yield: 75 mg (30%); *R*_f_ = 0.31 (10% MeOH in CH_2_Cl_2_); **LC‐MS** (ESI) (90% H_2_O to 100% MeCN in 10 min, then 100% MeCN to 15 min, DAD 220–600 nm), *t*_R_ = 5.23 min, 88% purity, *m*/*z* [M + H]^+^ calcd for C_28_H_39_N_4_O_5_, 511.29; found, 511.5; **HRMS** (ESI) *m*/*z* [M + H]^+^ calcd for C_28_H_39_N_4_O_5_, 511.2915; found, 511.2927.

##### 4‐Bromo‐N‐(2,6‐Dioxopiperidin‐3‐yl)‐2‐Fluorobenzamide (11a)

This compound was prepared by analogy with our previous method.^[^ ^19^ ^]^ In brief, 4‐bromo‐2‐fluorobenzoic acid(1.10 g, 5.0 mmol), 3‐aminopiperidine‐2,6‐dione hydrochloride (3.29 g, 20 mmol), and DIPEA (3.48 mL, 20 mmol) were suspended in dry DMF (20 mL). Subsequently, EDC × HCl (1.05 g, 5.5 mmol), and HOBt × H_2_O (0.84 g, 5.5 mmol) were added. After stirring the mixture for 18 h at rt, it was quenched by the addition of half‐saturated NH_4_Cl solution (100 mL) and then extracted with 10% MeOH in EtOAc (2 × 100 mL). The combined organic layers were washed with H_2_O and brine (each 100 mL), dried over Na_2_SO_4_, filtered, and concentrated in vacuo. After evaporation of the solvent, the crude product was purified with FC (gradient from 0% to 5% MeOH in CH_2_Cl_2_) to get the desired compound as a white solid. Yield: 1.27 g (77%); m.p. 232–234 °C; *R*_f_ = 0.46 (5% MeOH in CH_2_Cl_2_); **LC‐MS** (ESI) (90% H_2_O to 100% MeCN in 10 min, then 100% MeCN to 15 min, DAD 220–600 nm), *t*_R_ = 4.22 min, 99% purity, *m*/*z* [M + H]^+^ calcd for C_12_H_11_^79^BrFN_2_O_3_, 328.99; found, 329.1; **HRMS** (ESI) *m*/*z* [M + H]^+^ calcd for C_12_H_11_^79^BrFN_2_O_3_, 328.9932; found, 328.9931.

##### Tert‐Butyl (5‐(4‐((2,6‐Dioxopiperidin‐3‐yl)carbamoyl)‐3‐Fluorophenyl)pentyl)carbamate (11d)

This compound was prepared using the General Procedure II (0.50 mmol scale), NHP‐ester **5d** (0.21 g), and aryl bromide **11a** (0.16 g). After evaporation of the solvent, the crude product was purified with FC (gradient from 0 to 50% EtOAc in cyclohexane) to get the desired compound as a white solid. Yield: 0.13 g (53%); m.p. 125–127 °C; *R*_f_ = 0.33 (60% EtOAc in petroleum ether); **LC‐MS** (ESI) (90% H_2_O to 100% MeCN in 10 min, then 100% MeCN to 15 min, DAD 220–600 nm), *t*_R_ = 6.15 min, 97% purity, *m*/*z* [M + H − Boc]^+^ calcd for C_22_H_31_FN_3_O_5_, 336.17; found, 336.2; **HRMS** (ESI) *m*/*z* [M + H]^+^ calcd for C_22_H_31_FN_3_O_5_, 436.2242; found, 436.2243.

##### 5‐Bromo‐N‐(2,6‐Dioxopiperidin‐3‐yl)picolinamide (12a)

This compound was prepared by analogy with our previous method.^[^ ^19^ ^]^ In brief, 5‐bromopicolinic acid (0.40 g, 2.0 mmol), 3‐aminopiperidine‐2,6‐dione hydrochloride (1.32 g, 8.0 mmol), and DIPEA (1.39 mL, 8.0 mmol) were suspended in dry DMF (10 mL). Subsequently, EDC × HCl (0.42 g, 2.2 mmol) and HOBt × H_2_O (0.34 g, 2.2 mmol) were added. After stirring the mixture for 18 h at rt, it was quenched by the addition of half‐saturated NH_4_Cl solution (100 mL) and then extracted with 10% MeOH in EtOAc (2 × 100 mL). The combined organic layers were washed with H_2_O and brine (each 100 mL), dried over Na_2_SO_4_, filtered, and concentrated in vacuo. After evaporation of the solvent, the crude product was purified with FC (gradient from 20% to 80% EtOAc in cyclohexane) to get the desired compound as a blue solid. Yield: 0.46  g (74%); m.p. 214–218 °C; *R*_f_ = 0.73 (EtOAc); **LC‐MS** (ESI) (90% H_2_O to 100% MeCN in 10 min, then 100% MeCN to 15 min, DAD 220–600 nm), *t*_R_ = 3.70 min, 98% purity, *m*/*z* [M + H]^+^ calcd for C_11_H_11_^79^BrN_3_O_3_, 312.00; found, 311.9; **HRMS** (ESI) *m*/*z* [M + H]^+^ calcd for C_11_H_11_^79^BrN_3_O_3_, 311.9978; found, 311.9977.

##### Tert‐Butyl (5‐(6‐((2,6‐Dioxopiperidin‐3‐yl)carbamoyl)pyridin‐3‐yl)pentyl)carbamate (12d)

This compound was prepared using the General Procedure II (0.50 mmol scale), NHP‐ester **5d** (0.21 g), and aryl bromide **12a** (0.16 g). After evaporation of the solvent, the crude product was purified twice with FC (first, gradient from 20% to 80% EtOAc in cyclohexane, second from 0% to 10% DCM in MeOH) to get the desired compound as a yellowish oil. Yield: 50 mg (11%); *R*_f_ = 0.50 (EtOAc); **LC‐MS** (ESI) (90% H_2_O to 100% MeCN in 10 min, then 100% MeCN to 15 min, DAD 220–600 nm), *t*_R_ = 4.6 min, 96% purity, *m*/*z* [M + H]^+^ calcd for C_21_H_31_N_4_O_5_, 419.23; found, 419.4.

##### 4‐Bromo‐N‐(2,6‐Dioxopiperidin‐3‐yl)‐2‐Fluorobenzene‐Sulfonamide (13a)

To a solution of benzensulfonyl chloride derivative (1 eq) in THF (0.25 M) was added 3‐aminopiperidine‐2,6‐dione hydrochloride (1 eq) and K_2_CO_3_ (4 eq) at rt. The reaction mixture was stirred at 30 °C for 24 h. The reaction was quenched with H_2_O, extracted with EtOAc (3 × 20 mL/1.0 mmol), saturated NaCl solution, dried over Na_2_SO_4_, and filtered. After evaporation of the solvent, the crude product was purified with FC (gradient from 0% to 2% MeOH in CH_2_Cl_2_) to get the desired compound as a gray solid. Yield: 0.18 g (17%); m.p. 200 °C (decomp.); *R*_f_ = 0.21 (2% MeOH in CH_2_Cl_2_); **LC‐MS** (ESI) (90% H_2_O to 100% MeCN in 10 min, then 100% MeCN to 15 min, DAD 220–600 nm), *t*_R_ = 4.56 min, 96% purity, *m*/*z* [M + H]^+^ calcd for C_11_H_11_^79^BrFN_2_O_4_S, 364.96; found, 364.7; **HRMS** (ESI) *m*/*z* [M + H]^+^ calcd for C_11_H_11_^79^BrFN_2_O_4_S, 364.9601; found, 364.9559.

##### Tert‐Butyl (5‐(4‐(N‐(2,6‐Dioxopiperidin‐3‐yl)sulfamoyl)‐3‐Fluorophenyl)pentyl)carbamate (13d)

This compound was prepared using the General Procedure II (0.50 mmol scale), NHP‐ester **5d** (0.21 g), and aryl bromide **13a** (0.18 g). After evaporation of the solvent, the crude product was purified with FC (gradient from 0% to 5% MeOH in CH_2_Cl_2_) to get the desired compound as a colorless solid. Yield: 0.19 g (81%); m.p. 92–95 °C; *R*_f_ = 0.62 (60% EtOAc in petroleum ether); **LC‐MS** (ESI) (90% H_2_O to 100% MeCN in 10 min, then 100% MeCN to 15 min, DAD 220–600 nm), *t*_R_ = 6.46 min, 98% purity, *m*/*z* [M + H − Boc]^+^ calcd for C_16_H_22_FN_3_O_4_S, 372.14; found, 372.4; **HRMS** (ESI) *m*/*z* [M + H]^+^ calcd for C_21_H_31_FN_3_O_6_S, 472.1912; found, 472.1896.

##### 2‐(4‐(Morpholinomethyl)phenyl)acetic Acid (14)

Methyl 2‐(4‐(morpholinomethyl)phenyl)acetate (3.0 mmol, 0.75 g) was dissolved in THF (8 mL) and stirred with 2 M NaOH solution (3.7 mL) for 3 h. The reaction was acidified with 2N HCl until pH 6. Then, the reaction mixture was evaporated, dissolved in EtOH/CH_2_Cl_2_ mixture, filtered off, evaporated again, and purified with FC (gradient from 0% to 15% MeOH in CH_2_Cl_2_) to get the desired compound as a colorless oil. Yield: 0.39 g (55%); *R*_f_ = 0.62 (60% EtOAc in petroleum ether); **LC‐MS** (ESI) (90% H_2_O to 100% MeCN in 10 min, then 100% MeCN to 15 min, DAD 220–600 nm), *t*_R_ = 0.44 min, 95% purity, *m*/*z* [M + H]^+^ calcd for C_13_H_17_NO_3_, 236.13; found, 236.0.

##### 1,3‐Dioxoisoindolin‐2‐yl 2‐(4‐(morpholinomethyl)phenyl) Acetate (15)

This compound was prepared using the General Procedure I (3 mmol scale) and **14** (0.71 g). After evaporation of the solvent, a yellow solid was washed with EtOAc and used without further purification. Yield: 0.34 g (30%); m.p. 190–194 °C; *R*_f_ = 0.33 (80% EtOAc in petroleum ether); **LC‐MS** (ESI) (90% H_2_O to 100% MeCN in 10 min, then 100% MeCN to 15 min, DAD 220–600 nm), *t*_R_ = 6.31 min, 89% purity, *m*/*z* [M + H]^+^ calcd for C_21_H_20_N_2_O_5_, 380.14; found, 381.2.

##### 2‐(4‐Chlorophenyl)‐N‐((1‐(2,6‐Dioxopiperidin‐3‐yl)‐2‐Oxo‐1,2‐Dihydrobenzo[cd]indol‐6‐yl)methyl)‐2,2‐Difluoroacetamide (16)

The deprotected amine **8b** (0.05 g, 0.13 mmol), 2‐(4‐chlorophenyl)‐2,2‐difluoroacetic acid (0.03 g, 0.13 mmol), DMAP (0.02 g, 0.13 mmol), and HOBt (0.02 g, 0.14 mmol) were suspended in CH_2_Cl_2_ (3 mL) and DMA (3 mL). Then, EDC*HCl (0.04 g, 0.17 mmol) in CH_2_Cl_2_ (2 mL) was added dropwise. The reaction mixture was heated up to 50 °C for 6 h. After evaporation of the solvent, the crude product was purified with reversed phase FC (gradient from 10% to 90% CH_3_CN in H_2_O) to get the desired compound as a yellow solid. Yield: 0.03 g (45%); *R*_f_ = 0.26 (5% MeOH in CH_2_Cl_2_); **LC‐MS** (ESI) (90% H_2_O to 100% MeCN in 10 min, then 100% MeCN to 15 min, DAD 220–600 nm), *t*_R_ = 6.43 min, 99% purity, *m*/*z* [M + H]^+^ calcd for C_25_H_18_ClF_2_N_3_O_4_, 498.10; found, 498.0; **HRMS** (ESI) *m*/*z* [M + H]^+^ calcd for C_25_H_18_ClF_2_N_3_O_4_, 498,1027; found, 498.1028.

##### General Procedure III for the N‐Boc Deprotection

The Boc‐protected amine was stirred for 2 h at rt in a solution of dry CH_2_Cl_2_ and TFA (each 5 mL/0.5 mmol, respectively). The solvent was removed under vacuum, and the remaining residues were coevaporated with dry CH_2_Cl_2_ (3 × 10 mL). The residue was further dried under a high vacuum for at least 1 h.

##### General Procedure IV for HATU‐Mediated Amide Bond Formation Reactions

The deprotected acid was dissolved in dry DMF (5 mL/0.12 mmol) and DIPEA (4.0 eq). After adding HATU (1.1 eq), the mixture was stirred at rt for 10 min. The deprotected amine (1.0 eq) was dissolved in dry DMF (5 mL/0.12 mmol) and DIPEA (2.0 eq). After combining the two solutions, the mixture was stirred for 18 h at rt. The reaction was quenched with H_2_O (10 mL) and diluted with saturated NaHCO_3_ solution (30 mL/0.12 mmol) before extraction with EtOAc (3 × 50   mL/0.12 mmol) was performed. The combined organic layers were washed with 5% LiCl solution (3 × 50 mL) and saturated NaCl solution (50 mL), dried over Na_2_SO_4_, and filtered. Subsequently, the solvent was removed under vacuum.

##### 4‐(5‐(2‐((S)‐4‐(4‐Chlorophenyl)‐2,3,9‐Trimethyl‐6H‐Thieno[3,2‐f][1,2,4]triazolo[4,3‐a][1,4]diazepin‐6‐yl)acetamido)pentyl)‐N‐(2,6‐Dioxopiperidin‐3‐yl)‐2‐Fluorobenzamide (PROTAC 17)

This compound was prepared using the General Procedures **III** and **IV** (0.18 mmol scale), amine **11d** (80 mg), and JQ1 acid (70 mg). After evaporation of the solvent, the crude product was purified with chromatography (5% MeOH in CH_2_Cl_2_) to get the desired compound as a colorless solid. Yield: 0.06 g (49%); m.p. 144–146 °C; *R*_f_ = 0.49 (10% MeOH in CH_2_Cl_2_); **LC‐MS** (ESI) (90% H_2_O to 100% MeCN in 10 min, then 100% MeCN to 15 min, DAD 220–600 nm), *t*_R_ = 6.47 min, 99% purity, *m*/*z* [M + H]^+^ calcd for C_36_H_37_ClFN_7_O_4_S, 718.24; found, 718.4; **HRMS** (ESI) *m*/*z* [M + H]^+^ calcd for C_36_H_37_ClFN_7_O_4_S, 718.2373; found, 718.2364.

##### 5‐(5‐(2‐((S)‐4‐(4‐Chlorophenyl)‐2,3,9‐Trimethyl‐6H‐Thieno[3,2‐f][1,2,4]triazolo[4,3‐a][1,4]diazepin‐6‐yl)acetamido)pentyl)‐N‐(2,6‐Dioxopiperidin‐3‐yl)picolinamide (PROTAC 18)

This compound was prepared using the General Procedures **III** and **IV** (0.11 mmol scale), amine **11d** (50 mg), and JQ1 acid (40 mg). After evaporation of the solvent, the crude product was purified with chromatography (5% MeOH in CH_2_Cl_2_) to get the desired compound as a colorless solid. Yield: 30 mg (42%); m.p. 152–154 °C; *R*_f_ = 0.49 (10% MeOH in CH_2_Cl_2_); **LC‐MS** (ESI) (90% H_2_O to 100% MeCN in 10 min, then 100% MeCN to 15 min, DAD 220–600 nm), *t*_R_ = 6.25 min, 99% purity, *m*/*z* [M + H]^+^ calcd for C_35_H_37_ClN_8_O_4_S, 700.23; found, 701.2; **HRMS** (ESI) *m*/*z* [M + H]^+^ calcd for C_35_H_38_ClN_8_O_4_S, 701.2420; found, 701.2414.

##### 2‐((S)‐4‐(4‐Chlorophenyl)‐2,3,9‐Trimethyl‐6H‐Thieno[3,2‐f][1,2,4]triazolo[4,3‐a][1,4]diazepin‐6‐yl)‐N‐(5‐(4‐(N‐(2,6‐Dioxopiperidin‐3‐yl)sulfamoyl)‐3‐Fluorophenyl)pentyl)acetamide (PROTAC 19)

This compound was prepared using the General Procedures **III** and **IV** (0.3 mmol scale), amine **13d** (0.14 g), and JQ1 acid (0.12 g). After evaporation of the solvent, the crude product was purified with chromatography (5% MeOH in CH_2_Cl_2_) to get the desired compound as a white solid. Yield: 0.11 g (45%); m.p. 160–163 °C; *R*_f_ = 0.33 (10% MeOH in CH_2_Cl_2_); **LC‐MS** (ESI) (90% H_2_O to 100% MeCN in 10 min, then 100% MeCN to 15 min, DAD 220–600 nm), *t*_R_ = 6.65 min, 98% purity, *m*/*z* [M + H]^+^ calcd for C_35_H_38_ClFN_7_O_5_S_2_, 754.20; found, 754.1. **HRMS** (ESI) *m*/*z* [M + H]^+^ calcd for C_35_H_38_ClFN_7_O_5_S_2_, 754.2043; found, 754.2030.

##### 2‐((S)‐4‐(4‐Chlorophenyl)‐2,3,9‐Trimethyl‐6H‐Thieno[3,2‐f][1,2,4]triazolo[4,3‐a][1,4]diazepin‐6‐yl)‐N‐(5‐(2‐(2,6‐Dioxopiperidin‐3‐yl)‐1‐Oxoisoindolin‐5‐yl)pentyl)acetamide (PROTAC 20)

This compound was prepared using the General Procedures **III** and **IV**, amine **10d** (0.09 g) and JQ1 acid (0.08 g). After evaporation of the solvent, the crude product was purified with chromatography (10% MeOH in CH_2_Cl_2_) to get the desired compound as a white solid. Yield: 0.10 g (67%); m.p. 155 °C (decomp.); *R*_f_ = 0.44 (10% MeOH in CH_2_Cl_2_); **LC‐MS** (ESI) (90% H_2_O to 100% MeCN in 10 min, then 100% MeCN to 15 min, DAD 220–600 nm), *t*_R_ = 6.31 min, 99% purity, *m*/*z* [M + H]^+^ calcd for C_37_H_38_ClN_7_O_4_S, 712.25; found, 712.7; **HRMS** (ESI) *m*/*z* [M + H]^+^ calcd for C_37_H_38_ClN_7_O_4_S, 712.2467; found, 712.2488.

##### Computational Docking

For each compound, a 3D structure of one stereoisomer was generated before docking using LigPrep (Schrödinger Suite 2020‐2, Schrödinger, LLC, New York, NY, 2020). CRBN protein in complex with thalidomide (PDB: 4CI1) was prepared using Protein Preparation Wizard. Thalidomide was extracted, hydrogen atoms were added, residues were protonated at pH 7.4, the hydrogen bonding network was refined, and missing side chains were filled. The receptor's grid box was centered on the cocrystallized ligand thalidomide. Docking was performed with “Induced Fit” (Glide + PrimeX modules) scoring method.

##### Log*D*_7.4_ Measurements

The determination of the log*D*_7.4_ values was performed by a chromatographic method as described previously.^[^ ^29^ ^]^ Briefly, the system was calibrated by plotting the retention times of six different drugs (atenolol, metoprolol, labetalol, diltiazem, triphenylene, and permethrin) versus their literature known log*D*_7.4_ values to obtain a calibration line (*R* ^2^ ≥ 0.95). Subsequently, the mean retention times of the analytes were taken to calculate their log*D*_7.4_ values with the aid of the calibration line.

##### Plasma Protein Binding Studies^[^ ^30^ ^]^

HSA binding was estimated by correlating the logarithmic retention times of the analytes on a CHIRALPAK HSA 50 × 3 mm, 5 μm column with the literature known %PPB values (converted into log *K* values) of the following drugs: warfarin, ketoprofen, budesonide, nizatidine, indomethacin, acetylsalicylic acid, carbamazepine, piroxicam, nicardipine, and cimetidine. Samples were dissolved in MeCN/DMSO 9:1 to achieve a final concentration of 0.5 mg mL^–1^. Mobile phase A was 50 mM NH_4_OAc adjusted to pH 7.4 with ammonia, while mobile phase B was *i*PrOH. The flow rate was set to 1.0 mL min^–^^1^, the UV detector was set to 254 nm, and the column temperature was kept at 30 °C. After injecting 2 μL of the sample, a linear gradient from 100% A to 30% *i*PrOH in 5.4 min was applied. From 5.4 to 18 min, 30% *i*PrOH was kept, followed by switching back to 100% A in 1.0 min and a reequilibration time of 6 min. With the aid of the calibration line (*R* ^2^ ≥ 0.95), the log *K* values of new substances were calculated and converted to their %HSA values.

##### Competitive MST Assay

Competitive MST measurements were performed as described previously, using the human thalidomide binding domain (hTBD) and BODIPY‐uracil as a reporter.^[^ ^31^ ^]^ A 2 mM BODIPY‐uracil stock solution (in DMSO) was diluted 1:50 with MST‐buffer (50 mM TRIS pH 7.4, 150 mM NaCl, 10 mM MgCl_2_, 0.05% Tween‐20, and 1 mM TCEP) to prepare an intermediate stock. hTBD was diluted to 20 µM in MST‐buffer, and BODIPY‐uracil from the intermediate stock was added to 400 nM to generate the protein‐reporter mix. Compounds (dissolved in DMSO) were serially (16‐point 1:1) diluted in DMSO, and the resulting master dilution series was further diluted 1:100 with ddH_2_O to yield a final constant DMSO concentration of 0.5% (v/v). Mixing each, 5 µL of the protein‐reporter mix and the diluted compound a final concentration of 200 nM BODIPY‐uracil and 10 µM hTBD was achieved. Capillaries were loaded and the measurements were performed on a Monolith NT.115 with a Nano BLUE detector (NanoTemper Technologies) using 20% excitation power, medium MST power, and a temperature set to 25 °C. Data processing and conversion of IC_50_ values to *K*_i_ values were performed as described.^[^ ^31^ ^]^

##### Cell Viability Assay

5 × 10^3^ Molt4 cells were seeded in 50 µL RPMI (Gibco) medium supplemented with 10% fetal bovine serum (FBS) (Gibco) and 1% Pen‐Strep (Gibco) into white 384‐well plates (Corning, #3570), and compounds were dispensed using D300e Digital Dispenser (Tecan) and normalized to 0.1% DMSO. After 5 day incubation at 37 °C and 5% CO_2_, the cell viability was assessed. Therefore, the CTG assay was performed as described in the manufacturer's protocol (Promega, G7573). In brief, 12.5 µL/well premixed CTG reagent was added using ClipTip Pipettes (Thermo Fisher) and incubated for 10 min. The luminescence signal was quantified using PHERAstar FSX microplate reader (BMG Labtech). Data were analyzed using GraphPad Prism 10 software with curve fitting performed using a four‐parameter variable slope equation.

##### HiBiT‐BRD4 Degradation Assay^[^ ^32^ ^,^ ^33^ ^]^

HEK293T HiBiT degradation assays have been performed as previously described. In brief, HEK293T cells with a N‐terminal HiBiT knock in at the BRD4 locus (gift from Eric Fischer lab) were seeded at 10 × 10^3^ per well in a white 384‐well plate (Corning, #3570) in 50 µL/well DMEM media (Thermo Fisher Scientific, 41966‐029) supplemented with 10% FBS (Gibco) and 1% Pen‐Strep (Gibco). After overnight incubation at 37 °C and 5% CO_2_, compounds were dispensed using D300e Digital Dispenser (Tecan) and normalized to 0.1% DMSO. In the following, the assay plate was incubated at 37 °C and 5% CO_2_ for 5 h, and the HiBiT assay was performed as described in the manufacturer's protocol (Promega, N3030). Briefly, 12.5 µL/well premixed HiBiT reagent was added using ClipTip Pipettes (Thermo Fisher) and incubated for 10 min. The luminescence signal was quantified using PHERAstar FSX microplate reader (BMG Labtech). Data were analyzed using GraphPad Prism 10 software with curve fitting performed using a four‐parameter variable slope equation.

##### GSPT1 Degradation Assay

GSPT1 degradation assays have been performed as previously described. In brief, 30 × 10^3^ Flp293T GSPT1^389‐499^‐eGFP reporter cells (gift from Eric Fischer lab) per well were seeded in a 96‐well plate (Cellstar, 655 180) in 100 µL/well DMEM media (Thermo Fisher Scientific, 41966‐029) supplemented with 10% FBS (Gibco) and 1% Pen‐Strep (Gibco). After overnight incubation at 37 °C and 5% CO_2_, compounds were dispensed using D300e Digital Dispenser (Tecan) and normalized to 0.1% DMSO. After 5 h incubation at 37 °C and 5% CO_2_, the cells were trypsinized, resuspended in FACS buffer (PBS, 2% FBS, 1 mM EDTA), transferred to U‐bottom 96‐well plates (Greiner, # 650101), and analyzed by flow cytometer (guava easyCyte HT Luminex, Millipore). eGFP and mCherry fluorescence was measured from a minimum of 3000 signal events per well. Data was analyzed using FlowJo (FlowJo, LCC). Briefly, forward and side scatter were used to gate live cells, and a GFP to mCherry ratio for each cell was calculated. The mCherry‐positive cells were then selected to calculate the median of the eGFP/mCherry ratio. All ratios were normalized against the DMSO control. Data were analyzed using GraphPad Prism 10 with curve fitting performed using a four‐parameter variable slope equation.

##### SALL4A Degradation Reporter System

HEK293T cells were transiently transfected with Artichoke reporter vector (Addgene #73320) containing SALL4A. Cells were seeded into 96‐well plates and treated with 1 µM of respective compounds 48 h post‐transfection. After 16 h of treatment, cells were subjected to flow cytometry using the Beckman Coulter CytoFLEX S flow cytometer. Data analysis was performed with FlowJo v10.6.2. SALL4 protein levels were assessed by mean fluorescence intensity of SALL4‐GFP within the mCherry‐positive population. All values were normalized to the DMSO control.

##### Cellular CRBN NanoBRET Engagement Assay^[^ ^34^ ^]^

HEK293T stably expressing NanoLuc‐CRBN were cultured in DMEM (Gibco, Life Technologies) supplemented with 10% FBS. Cells were resuspended at 2 × 10^5^ viable cells/mL in 21 mL Opti‐MEM I (Gibco, Life Technologies) and mixed with 600 μL BODIPYTM‐lenalidomide fluorescent tracer (stock at 10 μM diluted in tracer dilution buffer 31.25% PEG‐400, 12.5 mM HEPES, pH 7.5, filtered using a 0.22 μm nitrocellulose membrane) to reach final concentration of the tracer at 278 nM. The cell–tracer mixture was then plated in a white polystyrene 384‐well plate (Corning, 3570) at 50 μL/well. After plating, the assay plate was centrifuged (400 × g, 5 min) and protected from light. Compounds for testing were added to the plate using a D300e Digital Dispenser (Tecan) in duplicate 12‐pt titrations from a 10 mM stock in DMSO, with DMSO normalized to 1% total volume. The plate was then placed in an incubator at 37 °C, 5% CO_2_ for 2 h. After incubation, the plate was removed and set on the bench to cool to room temperature (≈10 min). The NanoLuc substrate (500× solution, Promega Catalog number N2160 for 1000 assay kit) and extracellular inhibitor (1500× solution, Promega Catalog number N2160 for 1000 assay kit) were diluted in Opti‐MEM I (Gibco, Life Technologies) to prepare a 3× solution, which was added to each well (25 μL/well). The plate was read on a PHERAstar FSX (BMG Labtech) microplate reader with simultaneous dual emission capabilities to read 384‐well plates at 450 and 520 nm, for 10 cycles which were averaged to create data point. The NanoBRET ratio was calculated by dividing the signal at 520 nm by the signal at 450 nm and multiplying by 1000 for each sample. The data was plotted in GraphPad Prism 10, and the curves were fitted using Variable Slope equation to obtain the EC_50_ values.

## Conflict of Interest

The authors declare no conflict of interest.

## Supporting information

Supplementary Material

## Data Availability

The data that support the findings of this study are available in the Supporting Information of this article.
