# Supplementary material for: A Facile Protocol for C(sp2)–C(sp3) Bond Formation Reactions Toward Functionalized E3 Ligase Ligands
Source: ChemMedChem. 2025 Dec 16;21(4):e202500929. doi: 10.1002/cmdc.202500929 (PMC12913232; doi:10.1002/cmdc.202500929)
Supplement: Supplementary file 1 — Supplementary Material [file CMDC-21-e202500929-s001.pdf]

Supporting Information

A Facile Protocol for C(sp^2)–C(sp^3) Bond Formation Reactions Towards Functionalized Cereblon E3 ligase Ligands

Anita Maksutova,[a] Thomas M. Geiger,[b] Lorenzo Cianni,[b] Dominika E. Pieńkowska,[b] Jan Gerhartz,[b] Lina Read,[b] Aleša Bricelj,[c] Alexander Herrmann,[d,e] Maurice Leon Nelles,[a] Yuen Lam Dora Ng,[f] Marcus D. Hartmann,[d,e] Jan Krönke,[f,g] Izidor Sosič,[c] Radosław P. Nowak,[b] Michael Gütschow,[a] and Christian Steinebach*[a]

[a] Pharmaceutical Institute, University of Bonn, DE-53121 Bonn, Germany

[b] Institute of Structural Biology, University of Bonn, DE-53127 Bonn, Germany

[c] Faculty of Pharmacy, University of Ljubljana, SI-1000 Ljubljana, Slovenia

[d] Max Planck Institute for Biology, DE-72076 Tübingen, Germany

[e] Interfaculty Institute of Biochemistry, University of Tübingen, DE-72076 Tübingen, Germany.

[f] Charité – Universitätsmedizin Berlin, DE-12203 Berlin, Germany

[g] Universitätsmedizin Greifswald, DE-17475 Greifswald, Germany

E-mail: c.steinebach@uni-bonn.de

Table of Content

Supplementary Table...S3

Spectroscopic Details of Compounds **4** – **23** S4

Synthetic Procedures for Compounds **A** – **G**...S15

Selected NMR Spectra S18

References S24

Supplementary Table

Table S1. Binding Data, Physicochemical Properties, and Cellular Activities of Undecorated Cereblon Ligands.

**Cmpd**	IC_{50} (µM)[a]	K_i (µM)[b]	pIC_{50} (NanoBRET)[c]	elog$D_{7.4}$[d]	%PPB[e]	CHI_{IAM}[f]	log$S$[g]	dSALL4 (%)[h]	H_2DCFDA assay[j]	UV/Vis stability[h]	*in vitro* $t_{1/2}$ (min)
Thalidomide	23 ± 1.6	8.5 ± 0.8	5.78	0.5	36	12.5	–3.8	56	not active	< *p*H 8	>60
EM12	12.5 ± 0.7	3.1 ± 0.4	6.47	0.0	26	7.9	–3.3	63	not active	< *p*H 9	n.a.[l]
**A**	11.3 ± 0.8	2.5 ± 0.4	5.67	0.5	25	12.5	–2.3	<5	not active	n.a.	>60
**B**	127 ± 40[m]	63 ± 21	4.49	–0.3	10	6.3	n.d.	6	not active	< *p*H 9	>60
**C**	24.5 ± 1.8	9.3 ± 0.9	4.31	–0.3	11	2.8	n.d.	n.d.	not active	stable	n.d.
**D**	8.0 ± 0.6	n.a.[o]	7.06	0.4	24	11.9	n.d.	n.d.	not active	stable	n.a.
**E**	7.6 ± 0.3	n.a.[o]	8.31	1.2	84	24.4	n.d.	74	not active	stable	>60
**F**	43.3 ± 2.1	19.0 ± 1.1	5.26	0.8	49	16.1	n.d.	6	not active	n.a.	n.d.
**G**	29.1 ± 0.9	11.7 ± 0.5	5.23	0.4	18	13.3	n.d.	<5	active	< *p*H 9	n.a.

[a,b] Affinity values determined in a competitive MST assay as described in the Experimental Section (main manuscript). [c] CRBN engagement assay using NanoBRET technology. [d] Distribution coefficients at *p*H 7.4 were estimated by an HPLC-based method. [e] Plasma protein binding; experimentally determined percentage of compound bound to human serum albumin. [f] Chromatographic hydrophobicity index values referring to IAM chromatography (CHI_{IAM} values), an estimate for drug-membrane interactions and permeability. [g] Logarithm of the solubility measured in mol/L at *p*H 6.8 by an HPLC-based method. [h] Percentage of degraded SALL4 protein after 16 h treatment of HEK293T SALL4A reporter cells with 1 µM of each compound. Mean fluorescence intensity was obtained *via* flow cytometry, and values were normalized to the respective internal control and to DMSO-treated conditions. All data represent the average of at least two independent experiments. [j] Redox activity assays for the detection of compounds that react with 100 µM TCEP in redox cycles by forming ROS (H_2DCFDA assay) see Ref. 1. [k] UV-Vis-based assay for the evaluation of aqueous stability in phosphate buffer at *p*H 7.0, 8.0, and 9.0 after 4 h of incubation at 37 °C. [l] Not available due to spectral interference. [m] Not determined. [n] Data from Ref. 2. [o] Conversion of IC_{50} to K_i using Cheng-Prusoff equation is unreliable when the IC_{50} is in the same range as the reporter's K_i.

Spectroscopic Details of Compounds **4 – 23**

***rac*-Cemsisomide (4)**

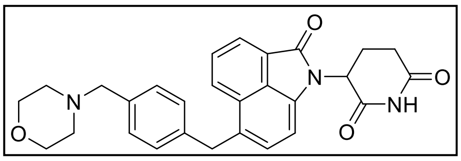

^{1}H NMR (600 MHz, DMSO-d_6) δ 2.04 – 2.11 (m, 1H), 2.28 (t, J = 4.5 Hz, 4H), 2.60 – 2.67 (m, 1H), 2.67 – 2.79 (m, 1H), 2.89 – 3.00 (m, 1H), 3.36 (s, 2H), 3.51 (t, J = 4.6 Hz, 4H), 4.36 (s, 2H), 5.42 (dd, J = 5.4, 13.0 Hz, 1H), 7.09 (d, J = 7.2 Hz, 1H), 7.18 (d, J = 7.9 Hz, 2H), 7.22 (d, J = 8.1 Hz, 2H), 7.38 (d, J = 7.3 Hz, 1H), 7.79 (dd, J = 7.0, 8.3 Hz, 1H), 8.06 (d, J = 7.0 Hz, 1H), 8.31 (d, J = 8.3 Hz, 1H), 11.09 (s, 1H); **^{13}C NMR** (151 MHz, DMSO-d_6) δ 22.54, 31.44, 36.69, 53.29, 62.30, 66.31, 124.30, 125.18, 125.85, 128.39, 128.48, 128.81, 129.01, 129.22, 129.42, 132.04, 135.62, 139.93, 167.02, 170.38, 172.97.

1,3-Dioxoisoindolin-2-yl (*tert*-butoxycarbonyl)glycinate (5b)

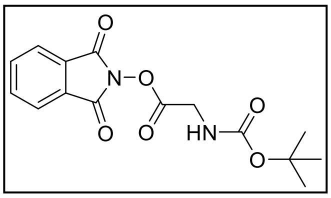

^{1}H NMR (500 MHz, DMSO-d_6) δ 1.39 (s, 9H), 4.18 (d, J = 6.2 Hz, 2H), 7.50 (t, J = 6.2 Hz, 1H), 7.92 – 7.99 (m, 4H); **^{13}C NMR** (126 MHz, DMSO-d_6) δ 28.23, 78.99, 124.16, 128.31, 135.68, 155.81, 161.74, 168.02.

1,3-Dioxoisoindolin-2-yl 3-((*tert*-butoxycarbonyl)amino)propanoate (5c)

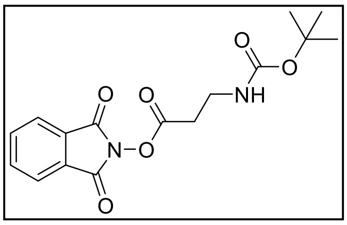

^{1}H NMR (500 MHz, $CDCl_3$) δ 1.42 (s, 10H), 2.88 (t, J = 6.0 Hz, 2H), 3.53 (q, J = 6.2 Hz, 2H), 5.06 – 5.16 (m, 1H), 7.75 – 7.80 (m, 2H), 7.84 – 7.89 (m, 2H); **^{13}C NMR** (126 MHz, $CDCl_3$) δ 28.32, 32.06, 36.09, 79.72, 124.02, 128.80, 134.83, 155.72, 161.80, 168.57.

1,3-Dioxoisoindolin-2-yl 6-((*tert*-butoxycarbonyl)amino)hexanoate (5d)

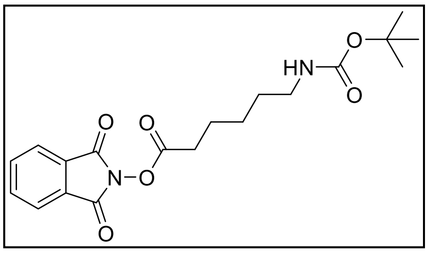

^{1}H NMR (500 MHz, DMSO-d_6) δ 1.32 – 1.47 (m, 14H), 1.66 (p, J = 7.4 Hz, 2H), 2.74 (t, J = 7.3 Hz, 2H), 2.92 (q, J = 6.5 Hz, 2H), 6.75 (t, J = 5.8 Hz, 1H), 7.92 – 7.99 (m, 4H); **^{13}C NMR** (126 MHz, DMSO-d_6) δ 24.12, 25.50, 28.41, 29.07, 30.26, 77.47, 124.12, 128.34, 135.67, 155.72, 162.03, 170.08.

1,3-Dioxoisoindolin-2-yl 7-((*tert*-butoxycarbonyl)amino)heptanoate (5e)

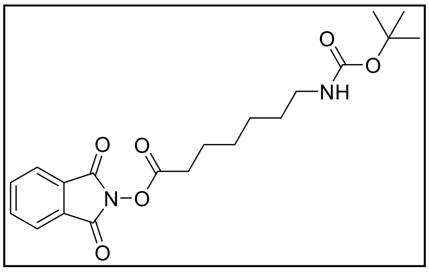

^{1}H NMR (500 MHz, DMSO-d_6) δ 1.22 – 1.31 (m, 3H), 1.32 – 1.42 (m, 13H), 1.64 (p, *J* = 7.4 Hz, 2H), 2.73 (t, *J* = 7.2 Hz, 2H), 2.90 (q, *J*= 6.5 Hz, 2H), 7.90 – 8.00 (m, 4H); **^{13}C NMR** (126 MHz, DMSO-d_6) δ 24.41, 25.92, 27.87, 28.41, 29.38, 30.23, 77.42, 124.11, 128.34, 135.65, 155.72, 162.00, 170.11.

***Tert*-Butyl 4-(3-((1,3-dioxoisoindolin-2-yl)oxy)-3-oxopropyl)piperidine-1-carboxylate (5f)**

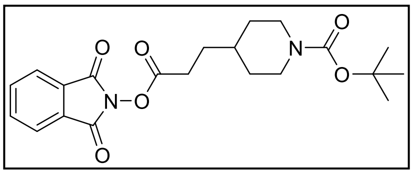

^{1}H NMR (500 MHz, DMSO-d_6) δ 1.00 (qd, *J* = 4.3, 12.4 Hz, 2H), 1.38 (s, 9H), 1.45 – 1.55 (m, 1H), 1.61 (q, *J* = 7.3 Hz, 2H), 1.64 – 1.70 (m, 2H), 2.58 – 2.72 (m, 2H), 2.77 (t, *J* = 7.5 Hz, 2H), 3.86 – 4.03 (m, 2H), 7.91 – 7.99 (m, 4H); **^{13}C NMR** (126 MHz, DMSO-d_6) δ 27.80, 28.23, 30.76, 31.32, 34.67, 78.57, 124.12, 128.33, 135.66, 153.97, 161.99, 170.26.

1-(*Tert*-Butyl) 4-(1,3-dioxoisoindolin-2-yl) piperidine-1,4-dicarboxylate (5g)

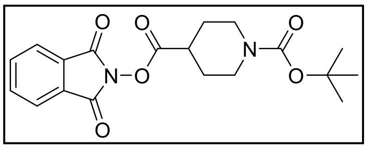

^{1}H NMR (500 MHz, DMSO-d_6) δ 1.39 (s, 9H), 1.50 – 1.63 (m, 2H), 1.92 – 2.02 (m, 2H), 2.91 – 3.04 (m, 2H), 3.09 – 3.17 (m, 1H), 3.80 – 3.91 (m, 2H), 7.95 (q, *J* = 4.9 Hz, 4H); **^{13}C NMR** (126 MHz, DMSO-d_6) δ 27.60, 28.18, 37.57, 79.01, 124.16, 128.34, 135.70, 153.98, 161.96, 171.24.

1'-(*Tert*-Butyl) 4-(1,3-dioxoisoindolin-2-yl) [1,4'-bipiperidine]-1',4-dicarboxylate (5h)

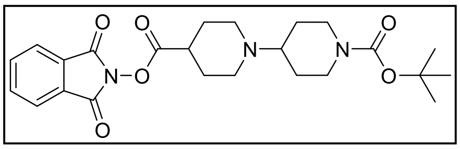

^{1}H NMR (600 MHz, DMSO-d_6) δ 1.39 (s, 9H), 1.48 – 1.63 (m, 2H), 1.98 – 2.30 (m, 6H), 2.60 – 2.87 (m, 2H), 2.91 – 3.14 (m, 2H), 3.17 – 3.28 (m, 1H), 3.40 – 3.60 (m, 2H), 3.90 – 4.21 (m, 2H), 7.93 – 8.00 (m, 4H); **^{13}C NMR** (151 MHz, DMSO-d_6) δ 25.19, 25.91, 28.18, 36.02, 47.34, 55.05, 62.61, 79.17, 124.22, 128.30, 135.78, 153.74, 161.90, 170.54.

***rac*-6-(*Tert*-Butyl) 1-(1,3-dioxoisoindolin-2-yl) 6-azaspiro[2.5]octane-1,6-dicarboxylate (5j)**

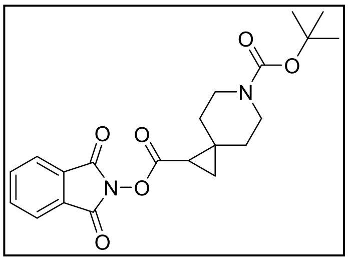

^{1}H NMR (500 MHz, $CDCl_3$) δ 1.22 (dd, *J* = 4.9, 8.0 Hz, 1H), 1.34 (t, *J* = 5.2 Hz, 1H), 1.36 – 1.42 (m, 1H), 1.45 (s, 9H), 1.59 – 1.65 (m, 1H), 1.66 – 1.76 (m, 2H), 1.88 (dd, *J* = 5.4, 8.0 Hz, 1H), 3.31 – 3.42 (m, 2H), 3.60 (dt, *J* = 5.0, 13.5 Hz, 1H), 3.63 – 3.70 (m, 1H), 7.74 – 7.78 (m, 2H), 7.83 – 7.88 (m, 2H); **^{13}C NMR** (126 MHz, $CDCl_3$) δ 21.65, 21.91, 28.25, 28.42, 31.65, 36.00, 79.56, 123.92, 128.90, 134.72, 154.71, 162.07, 168.48.

1-(*Tert*-Butyl) 3-(1,3-dioxoisoindolin-2-yl) azetidine-1,3-dicarboxylate (5k)

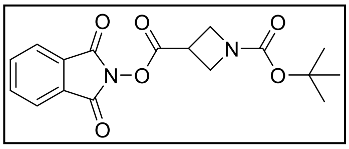

^{1}H NMR (500 MHz, $CDCl_3$) δ 1.41 (s, 1H), 1.43 (s, 9H), 4.22 – 4.30 (m, 4H), 7.77 – 7.82 (m, 2H), 7.86 – 7.90 (m, 2H); **^{13}C NMR** (126 MHz, $CDCl_3$) δ 28.29, 29.59, 80.32, 124.10, 128.78, 134.92, 155.76, 161.65, 169.02.

***Tert*-Butyl 4-(3-(2,4-dioxotetrahydropyrimidin-1(2*H*)-yl)phenyl)-[1,4'-bipiperidine]-1'-carboxylate (6h)**

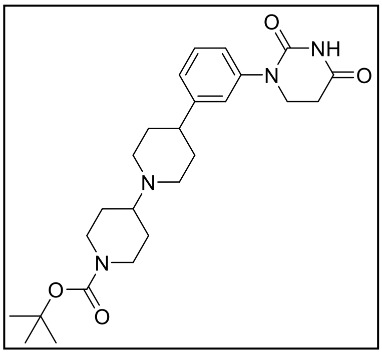

^{1}H NMR (500 MHz, DMSO-d_6) δ 10.32 (s, 1H), 7.32 (t, J = 7.8 Hz, 1H), 7.23 – 7.15 (m, 2H), 7.10 (d, J = 7.7 Hz, 1H), 4.03 (d, J = 12.1 Hz, 3H), 3.77 (t, J = 6.6 Hz, 2H), 2.89 – 2.63 (m, 7H), 2.04 – 1.71 (m, 7H), 1.44 – 1.35 (m, 11H); **^{13}C NMR** (126 MHz, DMSO-d_6) δ 23.19, 26.42, 28.16, 31.21, 44.73, 49.02, 62.38, 79.01, 123.47, 123.72, 124.05, 128.81, 142.31, 152.22, 153.79, 170.67.

3-(5-Bromo-3-methyl-2-oxo-2,3-dihydro-1*H*-benzo[*d*]imidazol-1-yl)piperidine-2,6-dione (7a)

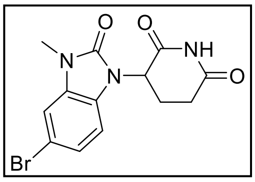

^{1}H NMR (600 MHz, DMSO-d_6) δ 1.96 – 2.05 (m, 1H), 2.58 – 2.73 (m, 2H), 2.83 – 2.92 (m, 1H), 3.33 (s, 3H), 5.36 (dd, J = 5.4, 13.0 Hz, 1H), 7.09 (d, J = 8.4 Hz, 1H), 7.20 (dd, J = 1.9, 8.4 Hz, 1H), 7.45 (d, J = 1.9 Hz, 1H), 11.09 (br s, 1H); **^{13}C NMR** (151 MHz, DMSO-d_6) δ 22.04, 27.30, 31.25, 51.96, 110.35, 111.16, 113.22, 123.47, 127.67, 131.43, 153.29, 169.88, 172.80.

3-(6-Bromo-2-oxobenzo[*cd*]indol-1(2*H*)-yl)piperidine-2,6-dione (8a)

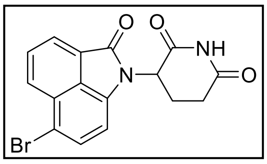

^{1}H NMR (600 MHz, DMSO-d_6) δ 2.14 – 2.07 (m, 1H), 2.68 – 2.61 (m, 1H), 2.79 – 2.69 (m, 1H), 2.98 – 2.89 (m, 1H), 5.45 (dd, J = 5.4, 13.1 Hz, 1H), 7.11 (d, J = 7.7 Hz, 1H), 7.82 (d, J = 7.7 Hz, 1H), 7.97 (t, J = 7.5 Hz, 1H), 8.18 (d, J = 3.5 Hz, 1H), 8.19 (d, J = 2.4 Hz, 1H), 11.12 (br s, 1H); **^{13}C NMR** (126 MHz, DMSO-d_6) δ 22.39, 31.39, 51.34, 108.25, 113.11, 125.66, 125.73, 125.87, 128.34, 130.28, 130.70, 131.76, 137.89, 166.52, 170.18, 172.90.

***Tert*-Butyl ((1-(2,6-dioxopiperidin-3-yl)-2-oxo-1,2-dihydrobenzo[*cd*]indol-6-yl)methyl)carbamate (8b)**

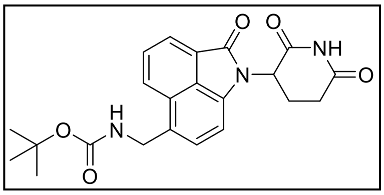

^{1}H NMR (600 MHz, DMSO-d_6) δ 1.38 (s, 9H), 2.05 – 2.12 (m, 1H), 2.61 – 2.68 (m, 1H), 2.70 – 2.80 (m, 1H), 2.92 – 2.99 (m, 1H), 4.54 (d, *J* = 5.9 Hz, 2H), 5.43 (dd, *J* = 5.5, 13.3 Hz, 1H), 7.08 (d, *J* = 7.3 Hz, 1H), 7.36 (d, *J* = 7.3 Hz, 1H), 7.46 (t, *J* = 6.1 Hz, 1H), 7.84 (t, *J* = 7.6 Hz, 1H), 8.09 (d, *J* = 6.9 Hz, 1H), 8.41 (d, *J* = 8.2 Hz, 1H), 11.10 (s, 1H); **^{13}C NMR** (151 MHz, DMSO-d_6) δ 22.53, 28.40, 31.45, 40.66, 51.15, 59.90, 78.03, 106.65, 124.35, 125.07, 125.74, 127.74, 128.98, 129.07, 130.46, 137.09, 155.74, 167.12, 170.34, 172.95.

***Tert*-Butyl (2-(1-(2,6-dioxopiperidin-3-yl)-2-oxo-1,2-dihydrobenzo[*cd*]indol-6-yl)ethyl)carbamate (8c)**

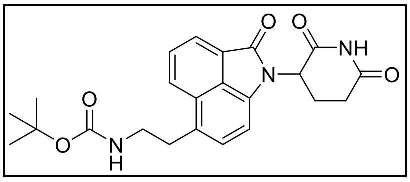

^{1}H NMR (500 MHz, DMSO-d_6) δ 1.36 (s, 9H), 2.03 – 2.12 (m, 1H), 2.61 – 2.68 (m, 1H), 2.70 – 2.80 (m, 1H), 2.90 – 3.00 (m, 1H), 3.09 – 3.15 (m, 2H), 3.22 (q, *J* = 6.8 Hz, 2H), 5.43 (dd, *J* = 5.4, 12.9 Hz, 1H), 6.93 (t, *J* = 5.7 Hz, 1H), 7.07 (d, *J* = 7.3 Hz, 1H), 7.31 (d, *J* = 7.3 Hz, 1H), 7.85 (t, *J* = 7.6 Hz, 1H), 8.09 (d, *J* = 7.0 Hz, 1H), 8.35 (d, *J* = 8.3 Hz, 1H), 11.09 (s, 1H); **^{13}C NMR** (126 MHz, DMSO-d_6) δ 22.55, 28.40, 31.43, 31.60, 41.95, 51.12, 77.71, 106.97, 124.21, 124.99, 125.87, 128.41, 128.64, 128.90, 128.94, 130.46, 136.43, 155.78, 167.00, 170.35, 172.94.

***Tert*-Butyl (5-(1-(2,6-dioxopiperidin-3-yl)-2-oxo-1,2-dihydrobenzo[*cd*]indol-6-yl)pentyl)carbamate (8d)**

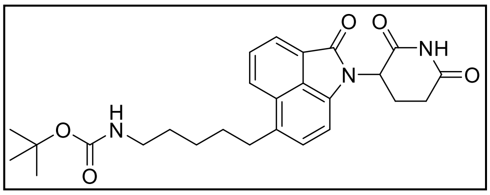

^{1}H NMR (500 MHz, DMSO-d_6) δ 11.09 (s, 1H), 8.32 (d, *J* = 8.2 Hz, 1H), 8.08 (d, *J* = 6.9 Hz, 1H), 7.83 (dd, *J* = 7.0, 8.3 Hz, 1H), 7.30 (d, *J* = 7.3 Hz, 1H), 7.04 (d, *J* = 7.2 Hz, 1H), 6.72 (t, *J* = 5.7 Hz, 1H), 5.42 (dd, *J* = 5.4, 13.0 Hz, 1H), 2.99 (t, *J* = 7.8 Hz, 2H), 2.96 – 2.92 (m, 1H), 2.90 (q, *J* = 6.4 Hz, 2H), 2.76 – 2.69 (m, 1H), 2.68 – 2.60 (m, 1H), 2.14 – 2.02 (m, 1H), 1.65 (p, *J* = 7.6 Hz, 2H), 1.42 (p, *J* = 6.9 Hz, 2H), 1.38 – 1.30 (m, 11H); **^{13}C NMR** (126 MHz, DMSO-d_6) δ 21.52, 22.58, 26.27, 28.41, 29.48, 31.02, 31.22, 31.43, 34.64, 37.59, 51.14, 77.43, 106.94, 124.17, 125.03, 125.93, 127.56, 128.38, 128.83, 129.03, 133.39, 136.09, 155.73, 167.02, 170.40, 172.96.

***Tert*-Butyl (6-(1-(2,6-dioxopiperidin-3-yl)-2-oxo-1,2-dihydrobenzo[*cd*]indol-6-yl)hexyl)carbamate (8e)**

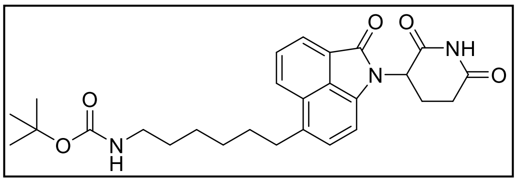

^{1}H NMR (500 MHz, DMSO-d_6) δ 1.36 (s, 15H), 1.65 (p, *J* = 7.5 Hz, 2H), 2.05 – 2.15 (m, 1H), 2.62 – 2.69 (m, 1H), 2.70 – 2.82 (m, 1H), 2.86 – 2.96 (m, 3H), 3.00 (t, *J* = 7.6 Hz, 2H), 5.43 (dd, *J* = 5.4, 13.0 Hz, 1H), 6.72 (t, *J* = 5.7 Hz, 1H), 7.06 (d, *J* = 7.3 Hz, 1H), 7.31 (d, *J* = 7.3 Hz, 1H), 7.84 (dd, *J* = 7.0, 8.3 Hz, 1H), 8.09 (d, *J* = 7.0 Hz, 1H), 8.32 (d, *J* = 8.2 Hz, 1H), 11.10 (s, 1H); **^{13}C NMR** (126 MHz, DMSO-d_6) δ 22.56, 26.25, 28.41, 28.74, 29.56, 30.96,

31.43, 31.52, 51.14, 77.41, 106.94, 124.15, 125.02, 125.94, 127.52, 128.36, 128.82, 128.96, 133.43, 136.07, 155.71, 167.01, 170.39, 172.94.

***Tert*-Butyl 4-(2-(1-(2,6-dioxopiperidin-3-yl)-2-oxo-1,2-dihydrobenzo[*cd*]indol-6-yl)ethyl)piperidine-1-carboxylate (8f)**

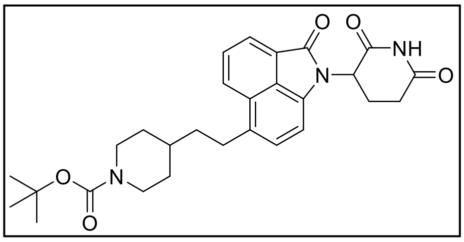

^{1}H NMR (500 MHz, DMSO-d_6) δ 1.01 – 1.12 (m, 2H), 1.39 (s, 9H), 1.46 – 1.54 (m, 1H), 1.60 (dt, J = 6.8, 9.7 Hz, 2H), 1.71 – 1.83 (m, 2H), 2.04 – 2.15 (m, 1H), 2.60 – 2.83 (m, 4H), 2.92 – 2.99 (m, 1H), 3.03 (dd, J = 4.1, 6.1 Hz, 2H), 3.90 – 3.98 (m, 2H), 5.43 (dd, J = 5.4, 13.0 Hz, 1H), 7.05 (d, J = 7.2 Hz, 1H), 7.32 (d, J = 7.4 Hz, 1H), 7.85 (dd, J = 7.0, 8.3 Hz, 1H), 8.09 (d, J = 7.0 Hz, 1H), 8.31 (d, J = 8.3 Hz, 1H), 11.10 (s, 1H); **^{13}C NMR** (126 MHz, DMSO-d_6) δ 22.54, 28.14, 28.25, 31.42, 31.80, 35.22, 38.37, 51.14, 78.51, 106.95, 124.18, 125.05, 125.95, 127.47, 128.27, 128.88, 133.40, 136.11, 153.98, 166.99, 170.38, 172.93.

***Tert*-Butyl 4-(1-(2,6-dioxopiperidin-3-yl)-2-oxo-1,2-dihydrobenzo[*cd*]indol-6-yl)piperidine-1-carboxylate (8g)**

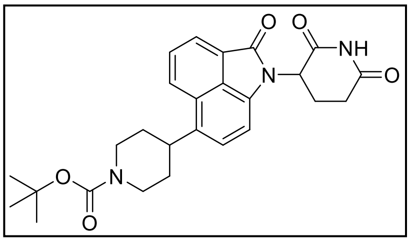

^{1}H NMR (500 MHz, DMSO-d_6) δ 1.43 (s, 9H), 1.58 – 1.69 (m, 2H), 1.82 – 1.91 (m, 2H), 2.05 – 2.15 (m, 1H), 2.62 – 2.69 (m, 1H), 2.70 – 2.82 (m, 1H), 2.88 – 3.07 (m, 3H), 3.50 (tt, J = 3.2, 12.1 Hz, 1H), 4.10 – 4.20 (m, 2H), 5.44 (dd, J = 5.4, 13.0 Hz, 1H), 7.09 (d, J = 7.5 Hz, 1H), 7.36 (d, J = 7.5 Hz, 1H), 7.83 – 7.89 (m, 1H), 8.10 (d, J = 7.0 Hz, 1H), 8.45 (d, J = 8.3 Hz, 1H), 11.10 (s, 1H); **^{13}C NMR** (126 MHz, DMSO-d_6) δ 22.55, 23.43, 28.28, 31.43, 33.11, 36.31, 51.11, 78.73, 107.07, 124.24, 124.70, 124.95, 126.00, 127.79, 128.54, 128.89, 136.76, 154.05, 167.03, 170.38, 172.94.

***Tert*-Butyl 4-(1-(2,6-dioxopiperidin-3-yl)-2-oxo-1,2-dihydrobenzo[*cd*]indol-6-yl)-[1,4'-bipiperidine]-1'-carboxylate (8h)**

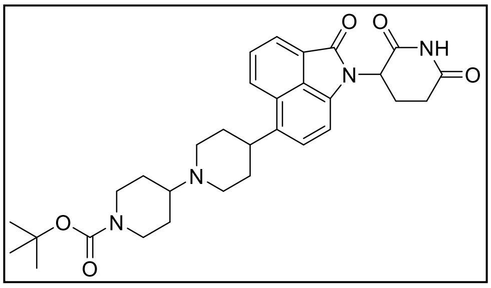

^{1}H NMR (600 MHz, DMSO-d_6) δ 1.31 – 1.37 (m, 1H), 1.39 (s, 9H), 1.77 (d, J = 12.3 Hz, 5H), 1.87 (d, J = 12.4 Hz, 2H), 2.04 – 2.11 (m, 1H), 2.61 – 2.67 (m, 1H), 2.70 – 2.80 (m, 2H), 2.90 – 2.98 (m, 1H), 3.02 (d, J = 10.7 Hz, 2H), 3.98 (d, J = 11.6 Hz, 2H), 5.43 (dd, J = 5.5, 13.1 Hz, 1H), 7.08 (d, J = 7.4 Hz, 1H), 7.36 (d, J = 7.5 Hz, 1H), 7.84 (t, J = 7.6 Hz, 1H), 8.08 (d, J = 6.9 Hz, 1H), 8.40 (d, J = 8.3 Hz, 1H), 11.09 (br s, 1H);

^{13}C NMR (151 MHz, DMSO-d_6) δ 22.56, 27.76, 28.25, 31.43, 33.60, 36.87, 49.55, 51.08, 61.52, 78.72, 107.13, 124.21, 124.52, 124.95, 125.99, 127.86, 128.57, 128.87, 136.08, 137.19, 154.01, 167.03, 170.40, 172.95.

***rac-Tert*-Butyl 1-(1-(2,6-dioxopiperidin-3-yl)-2-oxo-1,2-dihydrobenzo[*cd*]indol-6-yl)-6-aza-spiro[2.5] octane-6-carboxylate (8j)**

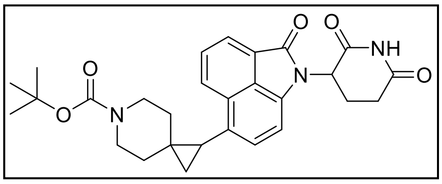

^{1}H NMR (600 MHz, DMSO-d_6) δ 1.26 – 1.47 (m, 12H), 1.85 – 1.93 (m, 1H), 2.05 – 2.13 (m, 1H), 2.44 (q, J = 7.0 Hz, 1H), 2.61 – 2.67 (m, 1H), 2.69 – 2.79 (m, 1H), 2.87 – 2.98 (m, 2H), 3.14 – 3.24 (m, 2H), 3.72 – 3.79 (m, 1H), 5.37 – 5.47 (m, 1H), 7.04 (t, J = 7.6 Hz, 1H), 7.19 (d, J = 7.4 Hz, 1H), 7.89 (t, J = 7.6 Hz, 1H), 8.10 (d, J = 7.0 Hz, 1H), 8.38 (dd, J = 2.4, 8.1 Hz, 1H), 11.09 (s, 1H); **^{13}C NMR** (151 MHz, DMSO-d_6) δ 15.45, 22.55, 24.62, 24.95, 24.99, 28.21, 31.43, 51.19, 78.64, 124.36, 125.02, 125.84, 127.13, 129.16, 129.34, 129.94, 130.11, 154.04, 167.01, 170.39, 172.96.

***Tert*-Butyl 3-(1-(2,6-dioxopiperidin-3-yl)-2-oxo-1,2-dihydrobenzo[*cd*]indol-6-yl)azetidine-1-carboxylate (8k)**

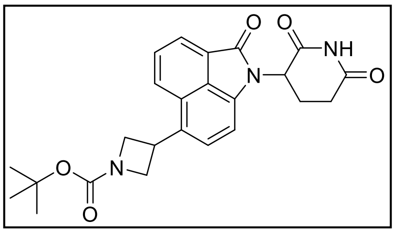

^{1}H NMR (500 MHz, DMSO-d_6) δ 1.40 (s, 9H), 2.05 – 2.14 (m, 1H), 2.61 – 2.68 (m, 1H), 2.70 – 2.81 (m, 1H), 2.90 – 3.01 (m, 1H), 3.99 – 4.07 (m, 2H), 4.36 – 4.52 (m, 3H), 5.44 (dd, J = 5.3, 13.0 Hz, 1H), 7.12 (d, J = 7.4 Hz, 1H), 7.51 (d, J = 7.4 Hz, 1H), 7.81 – 7.88 (m, 1H), 8.10 (d, J = 7.0 Hz, 1H), 8.19 (d, J = 8.2 Hz, 1H), 11.10 (br s, 1H); **^{13}C NMR** (126 MHz, DMSO-d_6) δ 14.22, 20.89, 22.52, 28.23, 29.61, 31.43, 51.22, 59.88, 78.88, 106.73, 124.52, 125.17, 125.62, 125.99, 127.66, 128.69, 129.23, 132.35, 136.91, 155.87, 167.02, 170.33, 172.93.

5-Bromo-2-(2,6-dioxopiperidin-3-yl)isoindoline-1,3-dione (9a)

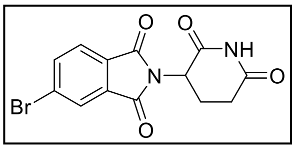

^{1}H NMR (400 MHz, DMSO-d_6) δ 2.01 – 2.12 (m, 1H), 2.53 – 2.70 (m, 2H), 2.82 – 2.96 (m, 1H), 5.17 (dd, J = 5.4, 12.9 Hz, 1H), 7.87 (dd, J = 0.6, 7.9 Hz, 1H), 8.10 (dd, J = 1.7, 7.9 Hz, 1H), 8.16 (dd, J = 0.5, 1.8 Hz, 1H), 11.15 (br s, 1H); **^{13}C NMR** (101 MHz, DMSO-d_6) δ 21.90, 30.91, 49.17, 125.30, 126.42, 128.55, 130.17, 133.18, 137.65, 165.90, 166.46, 169.71, 172.75.

***Tert*-Butyl 4-(2-(2,6-dioxopiperidin-3-yl)-1,3-dioxoisoindolin-5-yl)-[1,4'-bipiperidine]-1'-carboxylate (9h)**

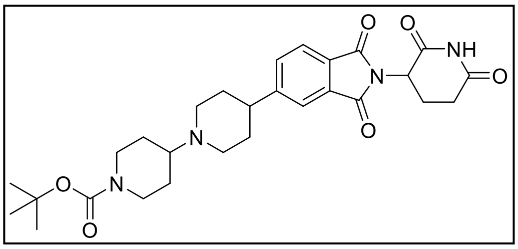

^{1}H NMR (600 MHz, DMSO-d_6) δ 0.92 (d, *J* = 7.4 Hz, 4H), 1.35 – 1.51 (m, 10H), 1.81 – 2.08 (m, 8H), 2.50 – 2.56 (m, 1H), 2.57 – 2.63 (m, 1H), 2.63 – 2.82 (m, 4H), 2.84 – 2.92 (m, 1H), 3.95 – 4.11 (m, 2H), 5.13 (dd, *J* = 5.4, 12.9 Hz, 1H), 7.73 – 7.81 (m, 2H), 7.87 (d, *J* = 7.6 Hz, 1H), 11.09 (br s, 1H); **^{13}C NMR** (151 MHz, DMSO-d_6) δ 22.11, 23.18, 28.17, 31.05, 49.11, 57.65, 78.97, 121.74, 123.85, 129.63, 131.93, 133.32, 153.80, 167.07, 167.24, 169.94, 172.84.

***Tert*-Butyl (5-(2-(2,6-dioxopiperidin-3-yl)-1-oxoisoindolin-5-yl)pentyl)carbamate (10d)**

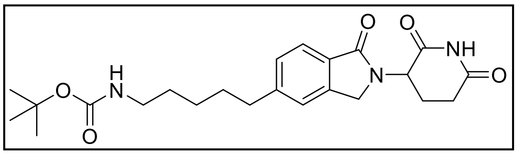

^{1}H NMR (500 MHz, DMSO-d_6) δ 1.22 – 1.30 (m, 2H), 1.36 (s, 9H), 1.38 – 1.44 (m, 2H), 1.60 (p, *J* = 7.6 Hz, 2H), 1.95 – 2.04 (m, 1H), 2.33 – 2.45 (m, 1H), 2.56 – 2.63 (m, 1H), 2.68 (t, *J* = 7.6 Hz, 2H), 2.86 – 2.96 (m, 3H), 4.29 (d, *J* = 17.1 Hz, 1H), 4.41 (d, *J* = 17.1 Hz, 1H), 5.09 (dd, *J* = 5.1, 13.3 Hz, 1H), 6.72 (t, *J* = 5.9 Hz, 1H), 7.33 (dd, *J* = 1.4, 7.8 Hz, 1H), 7.42 (s, 1H), 7.62 (d, *J* = 7.7 Hz, 1H), 10.95 (s, 1H); **^{13}C NMR** (126 MHz, DMSO-d_6) δ 22.68, 25.99, 28.42, 29.43, 30.75, 31.37, 35.48, 47.19, 51.70, 77.43, 122.95, 123.34, 128.43, 129.57, 142.55, 146.85, 155.72, 168.22, 171.22, 173.00.

***Tert*-Butyl 4-(2-(2,6-dioxopiperidin-3-yl)-1-oxoisoindolin-5-yl)-[1,4'-bipiperidine]-1'-carboxylate (10h)**

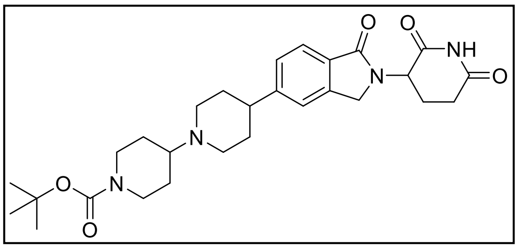

^{1}H NMR (500 MHz, DMSO-d_6) δ 1.35 – 1.43 (m, 9H), 1.87 (d, *J* = 23.6 Hz, 5H), 1.94 – 2.02 (m, 1H), 2.32 – 2.43 (m, 1H), 2.54 – 2.83 (m, 7H), 2.85 – 2.95 (m, 1H), 2.99 – 3.06 (m, 1H), 4.00 (d, *J* = 13.0 Hz, 2H), 4.29 (d, *J* = 17.2 Hz, 1H), 4.42 (d, *J* = 17.2 Hz, 1H), 5.08 (dd, *J* = 5.1, 13.3 Hz, 1H), 7.38 (d, *J* = 7.9 Hz, 2H), 7.47 (s, 1H), 7.65 (d, *J* = 7.9 Hz, 1H), 10.94 (s, 1H); **^{13}C NMR** (126 MHz, DMSO-d_6) δ 13.59, 19.33, 23.20, 28.20, 31.34, 47.27, 51.70, 52.19, 57.69, 62.72, 78.83, 121.79, 123.13, 126.93, 130.06, 142.64, 153.89, 168.10, 171.17, 172.97.

4-Bromo-*N*-(2,6-dioxopiperidin-3-yl)-2-fluorobenzamide (11a)

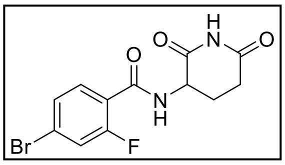

^{1}H NMR (500 MHz, DMSO-d_6) δ 1.96 – 2.14 (m, 2H), 2.48 – 2.57 (m, 1H), 2.72 – 2.83 (m, 1H), 4.70 – 4.79 (m, 1H), 7.53 (dd, *J* = 8.3, 1.8 Hz, 1H), 7.60 (t, *J* = 8.0 Hz, 1H), 7.68 (dd, *J* = 10.1, 1.9 Hz, 1H), 8.63 (dd, *J* = 8.3, 2.5 Hz, 1H), 10.84 (s, 1H); **^{13}C NMR** (126 MHz, DMSO-d_6) δ 24.11, 31.02, 49.85, 119.80 (d, *J* =

26.1 Hz), 122.80 (d, J = 13.8 Hz), 124.66 (d, J = 9.4 Hz), 127.98 (d, J = 3.9 Hz), 131.88 (d, J = 3.5 Hz), 159.28 (d, J = 254.5 Hz), 162.94, 171.84, 173.03.

***Tert*-Butyl (5-(4-((2,6-dioxopiperidin-3-yl)carbamoyl)-3-fluorophenyl)pentyl)carbamate (11d)**

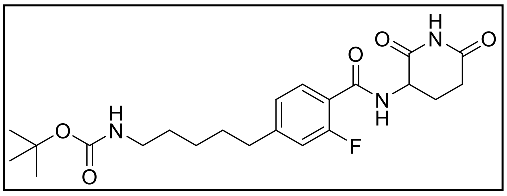

^{1}H NMR (600 MHz, DMSO-d_6) δ 1.20 – 1.28 (m, 2H), 1.36 (s, 11H), 1.57 (p, J = 7.7 Hz, 2H), 1.98 – 2.05 (m, 1H), 2.05 – 2.16 (m, 1H), 2.51 – 2.57 (m, 1H), 2.62 (t, J = 7.6 Hz, 2H), 2.74 – 2.83 (m, 1H), 2.89 (q, J = 6.6 Hz, 2H), 4.71 – 4.79 (m, 1H), 6.73 (t, J = 5.8 Hz, 1H), 7.10 – 7.16 (m, 2H), 7.58 – 7.64 (m, 1H), 8.43 (dd, J = 3.7, 8.2 Hz, 1H), 10.84 (s, 1H); **^{13}C NMR** (151 MHz, DMSO-d_6) δ 24.19, 25.91, 28.43, 30.17, 31.06, 34.72, 49.82, 77.46, 115.97 (d, J = 22.2 Hz), 120.42 (d, J = 13.3 Hz), 124.62 (d, J = 2.8 Hz), 130.37 (d, J = 3.2 Hz), 148.57 (d, J = 7.8 Hz), 155.74, 159.56 (d, J = 249.7 Hz), 163.58, 172.06, 173.09.

5-Bromo-*N*-(2,6-dioxopiperidin-3-yl)picolinamide (12a)

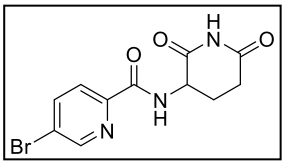

^{1}H NMR (500 MHz, DMSO-d_6) δ 1.95 – 2.04 (m, 1H), 2.15 – 2.27 (m, 1H), 2.54 (dd, J = 2.7, 4.5 Hz, 1H), 2.79 (ddd, J = 5.5, 13.7, 17.3 Hz, 1H), 4.77 (ddd, J = 5.3, 8.4, 12.5 Hz, 1H), 7.99 (d, J = 8.4 Hz, 1H), 8.26 (dd, J = 2.3, 8.4 Hz, 1H), 8.80 (d, J = 2.3 Hz, 1H), 9.07 (d, J = 8.4 Hz, 1H), 10.84 (s, 1H); **^{13}C NMR** (126 MHz, DMSO-d_6) δ 24.02, 31.10, 49.69, 123.80, 124.11, 140.72, 148.43, 149.47, 163.34, 172.10, 173.08.

***Tert*-Butyl (5-(6-((2,6-dioxopiperidin-3-yl)carbamoyl)pyridin-3-yl)pentyl)carbamate (12d)**

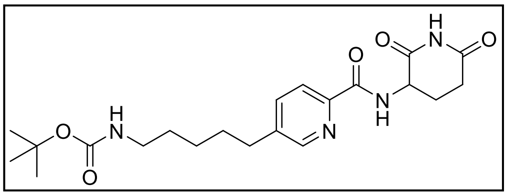

^{1}H NMR (600 MHz, DMSO-d_6) δ 1.21 – 1.43 (m, 4H), 1.35 (s, 9H), 1.52 – 1.63 (m, 3H), 1.96 – 2.04 (m, 1H), 2.15 – 2.25 (m, 1H), 2.67 (t, J = 7.6 Hz, 2H), 2.74 – 2.84 (m, 1H), 3.11 – 3.18 (m, 2H), 4.73 – 4.81 (m, 1H), 6.73 (t, J = 5.5 Hz, 1H), 7.82 (dd, J = 2.2, 7.9 Hz, 1H), 7.96 (d, J = 8.0 Hz, 1H), 8.50 (d, J = 2.0 Hz, 1H), 8.98 (d, J = 8.3 Hz, 1H), 10.83 (s, 1H); **^{13}C NMR** (151 MHz, DMSO-d_6) δ 13.64, 19.37, 21.55, 23.23, 24.16, 25.87, 28.43, 30.24, 31.15, 32.11, 34.65, 37.61, 49.60, 57.71, 77.46, 121.91, 137.44, 141.29, 148.60, 155.74, 164.07, 169.74, 172.31, 173.12.

4-Bromo-*N*-(2,6-dioxopiperidin-3-yl)-2-fluorobenzene-sulfonamide (13a)

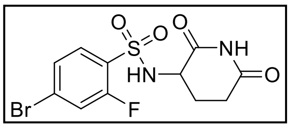

^{1}H NMR (500 MHz, DMSO-d_6) δ 1.97 – 1.84 (m, 2H), 2.48 – 2.43 (m, 1H), 2.77 – 2.64 (m, 1H), 4.30 – 4.19 (m, 1H), 7.57 (dd, J = 1.8, 8.4 Hz, 1H), 7.72 (t, J = 8.1 Hz, 1H), 7.79 (dd, J = 1.9, 9.8 Hz, 1H), 8.49 (s, 1H), 10.73 (br s, 1H);

^{13}C NMR (126 MHz, DMSO-d_6) δ 25.89, 30.73, 53.01, 120.47 (d, J = 24.6 Hz), 126.96 (d, J = 9.3 Hz), 127.81 (d, J = 3.8 Hz), 129.34 (d, J = 14.3 Hz), 130.68, 158.56 (d, J = 258.6 Hz), 171.61, 172.73.

***Tert*-Butyl (5-(4-(*N*-(2,6-dioxopiperidin-3-yl)sulfamoyl)-3-fluorophenyl)pentyl)carbamate (13d)**

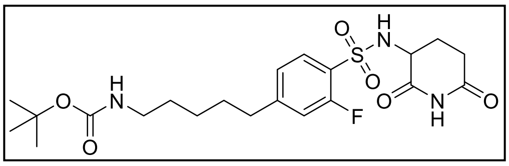

^{1}H NMR (500 MHz, DMSO-d_6) δ 1.20 – 1.27 (m, 2H), 1.35 (s, 11H), 1.56 (p, J = 7.6 Hz, 2H), 1.84 – 1.93 (m, 2H), 2.42 – 2.48 (m, 1H), 2.62 (t, J = 7.7 Hz, 2H), 2.65 – 2.74 (m, 1H), 2.88 (q, J = 6.6 Hz, 2H), 4.22 (t, J = 8.7 Hz, 1H), 6.72 (t, J = 5.9 Hz, 1H), 7.14 (dd, J = 1.6, 8.1 Hz, 1H), 7.23 (dd, J = 1.6, 11.4 Hz, 1H), 7.69 (t, J = 7.8 Hz, 1H), 8.24 (br s, 1H), 10.72 (br s, 1H); **^{13}C NMR** (126 MHz, DMSO-d_6) δ 25.94, 28.41, 29.34, 30.01, 30.68, 34.77, 52.98, 77.45, 116.66 (d, J = 21.2 Hz), 124.29, 127.15 (d, J = 14.4 Hz), 129.11, 134.46, 150.74 (d, J = 7.6 Hz), 155.72, 158.58 (d, J = 253.5 Hz), 171.50, 172.73.

2-(4-(Morpholinomethyl)phenyl)acetic acid (14)

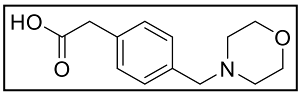

^{1}H NMR (600 MHz, DMSO-d_6) δ 2.35 (t, J = 4.6 Hz, 4H), 3.45 (s, 2H), 3.53 (s, 2H), 3.56 (t, J = 4.6 Hz, 4H), 7.21 (d, J = 8.1 Hz, 2H), 7.24 (d, J = 7.9 Hz, 2H); **^{13}C NMR** (151 MHz, DMSO-d_6) δ 40.54, 53.25, 62.23, 66.28, 129.05, 129.34, 133.89, 136.04, 172.83.

1,3-Dioxoisoindolin-2-yl 2-(4-(morpholinomethyl)phenyl)acetate (15)

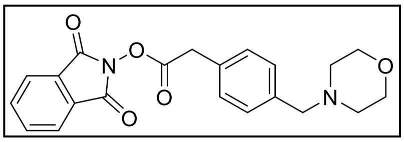

^{1}H NMR (500 MHz, DMSO-d_6) δ 8.02 – 7.90 (m, 4H), 7.64 (d, J = 7.8 Hz, 2H), 7.46 (d, J = 7.8 Hz, 2H), 4.33 – 4.23 (m, 6H), 3.60 (s, 2H), 3.21 – 3.11 (m, 4H).

2-(4-Chlorophenyl)-*N*-((1-(2,6-dioxopiperidin-3-yl)-2-oxo-1,2-dihydrobenzo[*cd*]indol-6-yl)methyl)-2,2-difluoroacetamide (16)

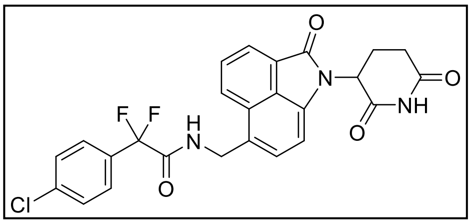

^{1}H NMR (500 MHz, DMSO-d_6) δ 11.11 (s, 1H), 9.61 (t, J = 5.8 Hz, 1H), 8.29 (d, J = 8.3 Hz, 1H), 8.10 (d, J = 7.0 Hz, 1H), 7.81 (t, J = 7.6 Hz, 1H), 7.58 (s, 4H), 7.38 (d, J = 7.4 Hz, 1H), 7.10 (d, J = 7.4 Hz, 1H), 5.44 (dd, J = 5.4, 13.2 Hz, 1H), 4.77 (d, J = 5.8 Hz, 2H), 3.00 – 2.90 (m, 1H), 2.83 – 2.69 (m, 1H), 2.69 – 2.60 (m, 1H), 2.14 – 2.08 (m, 1H); **^{13}C NMR** (126 MHz, DMSO-d_6) δ 172.95, 170.31, 167.08, 163.02 (t, J = 31.1 Hz), 137.53, 136.09, 132.13 (t, J = 26.2 Hz), 129.13, 129.09, 128.82, 128.63, 128.39, 127.65, 127.35

(t, J = 5.8 Hz), 125.75, 125.03, 124.50, 114.57 (t, J = 252.4 Hz), 106.59, 51.18, 31.44, 30.83, 22.50; **^{19}F NMR** (471 MHz, DMSO-d_6) δ –102.56.

4-(5-(2-((*S*)-4-(4-Chlorophenyl)-2,3,9-trimethyl-6*H*-thieno[3,2-*f*][1,2,4]triazolo[4,3-*a*][1,4]diazepin-6-yl)acetamido)pentyl)-*N*-(2,6-dioxopiperidin-3-yl)-2-fluorobenzamide (PROTAC 17)

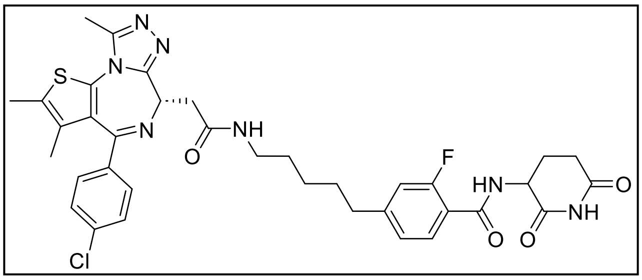

^{1}H NMR (500 MHz, DMSO-d_6) δ 1.25 – 1.39 (m, 2H), 1.43 – 1.54 (m, 2H), 1.55 – 1.69 (m, 5H), 1.94 – 2.17 (m, 2H), 2.41 (s, 3H), 2.52 – 2.65 (m, 6H), 2.67 – 2.85 (m, 1H), 3.02 – 3.15 (m, 2H), 3.16 – 3.25 (m, 1H), 4.51 (t, J = 7.0 Hz, 1H), 4.75 (p, J = 6.4 Hz, 1H), 7.13 (t, J = 8.0 Hz, 2H), 7.39 – 7.52 (m, 4H), 7.61 (t, J = 7.8 Hz, 1H), 7.95 (s, 1H), 8.16 (t, J = 5.7 Hz, 1H), 8.33 – 8.53 (m, 1H), 10.83 (s, 1H); **^{13}C NMR** (151 MHz, DMSO-d_6) δ 11.43, 12.83, 14.19, 26.10, 29.16, 30.23, 30.93, 31.06, 34.74, 37.84, 38.50, 49.82, 54.10, 115.92 (d, J = 22.2 Hz), 120.44 (d, J = 14.1 Hz), 124.57 (d, J = 4.0 Hz), 128.61, 129.74, 129.96, 130.27, 130.37 (d, J = 2.6 Hz), 130.88, 132.41, 135.40, 136.90, 148.56 (d, J = 7.7 Hz), 149.94, 155.29, 159.56 (d, J = 249.7 Hz), 163.12, 163.57, 169.49, 172.05, 173.09. **^{19}F NMR** (471 MHz, DMSO-d_6) δ -114.89 – -114.67 (m).

5-(5-(2-((*S*)-4-(4-Chlorophenyl)-2,3,9-trimethyl-6*H*-thieno[3,2-*f*][1,2,4]triazolo[4,3-*a*][1,4]diazepin-6-yl)acetamido)pentyl)-*N*-(2,6-dioxopiperidin-3-yl)picolinamide (PROTAC 18)

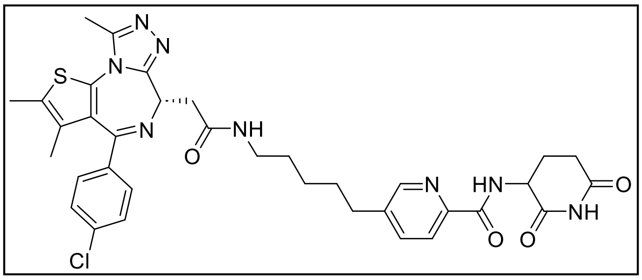

^{1}H NMR (600 MHz, DMSO-d_6) δ 10.84 (s, 1H), 8.98 (d, J = 8.4 Hz, 1H), 8.49 (d, J = 2.4 Hz, 1H), 8.16 (t, J = 5.7 Hz, 1H), 7.96 (d, J = 8.0 Hz, 1H), 7.82 (dd, J = 2.3, 8.0 Hz, 1H), 7.46 (d, J = 8.7 Hz, 2H), 7.41 (d, J = 8.3 Hz, 2H), 4.87 – 4.71 (m, 1H), 4.50 (dd, J = 6.1, 8.0 Hz, 1H), 3.26 – 3.16 (m, 2H), 3.16 – 3.05 (m, 2H), 2.84 – 2.74 (m, 1H), 2.67 (t, J = 7.7 Hz, 2H), 2.58 (s, 3H), 2.55 – 2.50 (m, 1H), 2.40 (s, 3H), 2.25 – 2.15 (m, 1H), 2.04 – 1.95 (m, 1H), 1.68 – 1.57 (m, 5H), 1.48 (p, J = 7.0 Hz, 2H), 1.37 – 1.30 (m, 2H); **^{13}C NMR** (151 MHz, DMSO-d_6) δ 11.44, 12.83, 14.20, 24.17, 26.08, 29.10, 30.29, 31.16, 32.13, 37.82, 38.49, 49.60, 54.08, 121.93, 128.61, 129.74, 129.96, 130.26, 130.88, 132.41, 135.40, 136.90, 137.39, 141.29, 147.51, 148.56, 149.94, 155.29, 163.13, 164.06, 169.49, 172.31, 173.12.

2-((*S*)-4-(4-Chlorophenyl)-2,3,9-trimethyl-6*H*-thieno[3,2-*f*][1,2,4]triazolo[4,3-*a*][1,4]diazepin-6-yl)-*N*-(5-(4-(*N*-(2,6-dioxopiperidin-3-yl)sulfamoyl)-3-fluorophenyl)pentyl)acetamide (PROTAC 19)

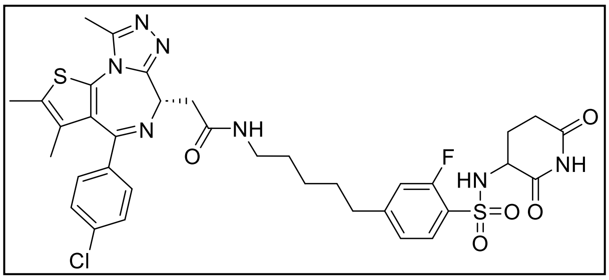

^{1}H NMR (600 MHz, DMSO-d_6) δ 1.33 (p, *J* = 7.6 Hz, 2H), 1.48 (p, *J* = 7.2 Hz, 2H), 1.56 – 1.66 (m, 5H), 1.89 (td, *J* = 4.3, 10.0 Hz, 2H), 2.41 (s, 3H), 2.46 (dt, *J* = 4.1, 17.5 Hz, 1H), 2.59 (s, 3H), 2.63 (t, *J* = 7.8 Hz, 2H), 2.67 – 2.75 (m, 1H), 3.11 (hept, *J* = 6.6 Hz, 2H), 3.22 (qd, *J* = 7.1, 15.0 Hz, 2H), 4.24 (q, *J* = 8.4 Hz, 1H), 4.51 (t, *J* = 7.0 Hz, 1H), 7.15 (d, *J* = 8.0 Hz, 1H), 7.24 (d, *J* = 11.3 Hz, 1H), 7.43 (d, *J* = 8.3 Hz, 2H), 7.48 (d, *J* = 8.2 Hz, 2H), 7.71 (t, *J* = 7.8 Hz, 1H), 8.14 – 8.19 (m, 1H), 8.27 (d, *J* = 7.7 Hz, 1H), 10.74 (s, 1H); **^{13}C NMR** (151 MHz, DMSO-d_6) δ 11.44, 12.83, 14.20, 25.95, 26.14, 29.12, 30.09, 30.69, 34.81, 37.82, 38.47, 52.98, 54.08, 116.63 (d, *J* = 20.9 Hz), 124.25, 127.18 (d, *J* = 14.3 Hz), 128.61, 129.13, 129.85 (d, *J* = 34.1 Hz), 130.27, 130.87, 132.41, 136.15 (d, *J* = 225.6 Hz), 149.94, 150.75 (d, *J* = 7.7 Hz), 155.29, 157.76, 159.44, 163.13, 169.49, 171.53, 172.75.

2-((*S*)-4-(4-Chlorophenyl)-2,3,9-trimethyl-6*H*-thieno[3,2-*f*][1,2,4]triazolo[4,3-*a*][1,4]diazepin-6-yl)-*N*-(5-(2-(2,6-dioxopiperidin-3-yl)-1-oxoisoindolin-5-yl)pentyl)acetamide (PROTAC 20)

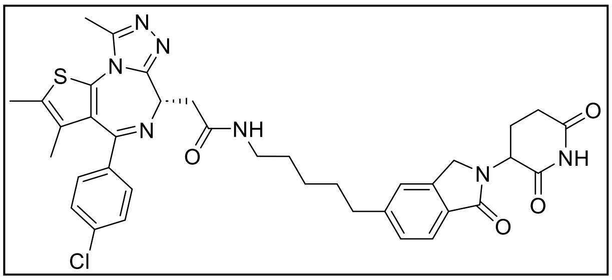

^{1}H NMR (600 MHz, DMSO-d_6) δ 1.34 (h, *J* = 6.7 Hz, 2H), 1.48 (p, *J* = 7.1 Hz, 2H), 1.56 – 1.66 (m, 5H), 1.93 – 2.02 (m, 1H), 2.32 – 2.42 (m, 4H), 2.56 – 2.61 (m, 4H), 2.67 (t, *J* = 7.7 Hz, 2H), 2.86 – 2.95 (m, 1H), 3.04 – 3.15 (m, 1H), 3.15 – 3.27 (m, 2H), 4.27 (dd, *J* = 6.9, 17.0 Hz, 1H), 4.40 (d, *J* = 17.0 Hz, 1H), 4.49 – 4.54 (m, 1H), 5.08 (dd, *J* = 5.1, 13.3 Hz, 1H), 7.31 (d, *J* = 7.8 Hz, 1H), 7.38 – 7.44 (m, 3H), 7.45 – 7.50 (m, 2H), 7.61 (d, *J* = 7.7 Hz, 1H), 8.16 (t, *J* = 5.7 Hz, 1H), 10.95 (s, 1H); **^{13}C NMR** (151 MHz, DMSO-d_6) δ 11.76, 13.15, 14.52, 23.00, 26.47, 29.51, 31.15, 31.70, 35.79, 38.16, 38.83, 47.50, 52.02, 54.42, 123.29, 123.63, 128.72, 128.93, 129.91, 130.06, 130.28, 130.58, 131.19, 132.74, 135.72, 137.22, 142.89, 147.18, 150.26, 155.62, 163.44, 168.54, 169.81, 171.55, 173.34.

Synthetic Procedures for Compounds **A – G**

Phenylglutarimide (A)

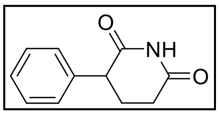

This compound was prepared by analogy with a previously reported method.[3] Yield: 34%; mp: 150–152 °C; R_f = 0.21 (30% EtOAc/cyclohexane); **^{1}H NMR** (500 MHz, DMSO-d_6) δ 1.99 – 2.08 (m, 1H), 2.12 – 2.24 (m, 1H), 2.44 – 2.52 (m, 1H), 2.60 – 2.71 (m, 1H), 3.84 (dd, J = 5.0, 11.5 Hz, 1H), 7.18 – 7.36 (m, 5H), 10.80 (br s, 1H); **^{13}C NMR** (126 MHz, DMSO-d_6) δ 26.10, 31.48, 47.48, 126.99, 128.43, 128.71, 139.32, 173.51, 174.35; **LC-MS** (ESI) (90% H_2O to 100% MeCN in 10 min, then 100% MeCN to 20 min, DAD 190-600 nm), t_R = 3.67 min, 99% purity, m/z $[M + H]^+$ calcd for $C_{11}H_{12}NO_2$, 190.09; found, 190.0.

***N*-(2,6-Dioxopiperidin-3-yl)benzamide (B)**

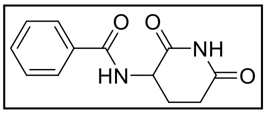

This compound was synthesized as we reported previously.[2]

1-Phenyldihydropyrimidine-2,4(1*H*,3*H*)-dione (C)

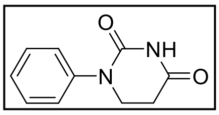

This compound was prepared by analogy with a previously reported method.[4] In brief, aniline (0.46 mL, 5 mmol) was suspended in acrylic acid (1.3 mL, 25 mmol), and the mixture was heated to 100 °C for 3 h. The reaction mixture was cooled to rt, acetic acid (5 mL) was added, and the mixture was heated to 100 °C for 10 minutes before urea (1.8 g, 30 mmol) was added. The reaction mixture was stirred at 120 °C for 18 h. After cooling to rt, the reaction mixture was poured onto ice cold HCl solution (1 mol/L, 20 mL) and stirred in an ice bath for 3 h. The resulting suspension was filtrated and dried to get a colorless solid. Yield: 0.60 g (63%); mp 186–188 °C; R_f = 0.41 (80% EtOAc in petroleum ether); **^{1}H NMR** (600 MHz, DMSO-d_6) δ 2.70 (t, J = 6.6 Hz, 2H), 3.78 (t, J = 6.7 Hz, 2H), 7.24 – 7.20 (m, 1H), 7.34 – 7.30 (m, 2H), 7.40 – 7.36 (m, 2H), 10.33 (s, 1H); **^{13}C NMR** (126 MHz, DMSO-d_6) δ 31.24, 44.71, 125.41, 125.93, 128.76, 142.23, 152.28, 170.73; **LC-MS** (ESI) (90% H_2O to 100% MeCN in 10 min, then 100% MeCN to 15 min, DAD 220-600 nm), t_R = 2.52 min, 97% purity, m/z $[M + H]^+$ calcd for $C_{10}H_{11}N_2O_2$, 191.08; found, 191.1.

3-(3-Methyl-2-oxo-2,3-dihydro-1*H*-benzo[*d*]imidazol-1-yl)piperidine-2,6-dione (D)

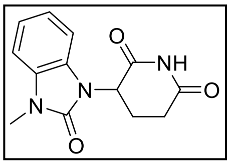

This compound was prepared by analogy with a previously reported method.[5] In brief, to a solution of NaH (60% dispersion in mineral oil, 62 mg, 1.54 mmol) in dry DMF (10 mL) was first added 1-methyl-1,3-dihydro-2*H*-benzo[*d*]imidazol-2-one (0.11 g, 0.77 mmol), and after 5 min, 3-bromopiperidine-2,6-dione (0.18 g, 0.93 mmol). The reaction mixture was stirred at 0 °C for 4 h. Subsequently, it was quenched with water (20 mL), neutralized with saturated NH_4Cl solution (10 mL), concentrated under reduced pressure, and purified by FC (EtOAc/cyclohexane/MeOH=4:5.5:0.5) to get the title compound as a colorless solid. Yield: 36 mg (18%); mp 228 °C (decomp.); R_f = 0.24 (EtOAc/petroleum ether/MeOH = 4/5.5/0.5); **^{1}H NMR** (600 MHz, DMSO-d_6) δ 2.06 – 1.97 (m, 1H), 2.67 – 2.59 (m, 1H), 2.77 – 2.66 (m, 1H), 2.95 – 2.84 (m, 1H), 3.34 (s, 3H), 5.37 (dd, *J* = 5.4, 13.0 Hz, 1H), 7.04 (t, *J* = 7.6 Hz, 1H), 7.09 (t, *J* = 7.7 Hz, 1H), 7.12 (d, *J* = 7.8 Hz, 1H), 7.18 (d, *J* = 7.7 Hz, 1H), 11.08 (br s, 1H); **LC-MS** (ESI) (90% H_2O to 100% MeCN in 10 min, then 100% MeCN to 15 min, DAD 220-600 nm), t_R = 3.41 min, 96% purity, *m*/*z* $[M + H]^+$ calcd for $C_{13}H_{14}N_3O_3$, 260.10; found, 260.1.

3-(2-Oxobenzo[*cd*]indol-1(2*H*)-yl)piperidine-2,6-dione (E)

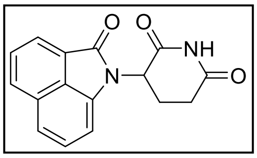

To a solution of NaH (60% dispersion in mineral oil, 0.24 g, 5.9 mmol) in DMF (20 mL) was first added benzo[*cd*]indol-2(1*H*)-one (0.51 g, 3.0 mmol), and after 15 min, 3-bromopiperidine-2,6-dione (0.68 g, 3.6 mmol). The reaction mixture was stirred at rt for 2 days. Subsequently, it was quenched with H_2O, neutralized with saturated NH_4Cl solution, extracted with EtOAc (3 × 25 mL), dried over Na_2SO_4, concentrated, and purified with FC (gradient from cyclohexane to cyclohexane/acetone/CH_2Cl_2=3:1:15) to get the desired compound as a yellow solid. Yield: 92 mg (11%); mp 242-243 °C; R_f = 0.23 (5% acetone in CH_2Cl_2); **^{1}H NMR** (500 MHz, DMSO-d_6) δ 2.15 – 2.05 (m, 1H), 2.70 – 2.61 (m, 1H), 2.82 – 2.70 (m, 1H), 3.00 – 2.90 (m, 1H), 5.45 (dd, *J* = 5.4, 13.0 Hz, 1H), 7.15 (d, *J* = 7.1 Hz, 1H), 7.53 (dd, *J* = 7.1, 8.5 Hz, 1H), 7.66 (d, *J* = 8.4 Hz, 1H), 7.83 (dd, *J* = 7.0, 8.2 Hz, 1H), 8.09 (d, *J* = 7.0 Hz, 1H), 8.22 (d, *J* = 8.1 Hz, 1H), 11.11 (br s, 1H); **^{13}C NMR** (126 MHz, DMSO-d_6) δ 22.53, 31.43, 51.22, 106.83, 120.41, 124.50, 124.66, 125.43, 128.95, 128.99, 129.17, 131.60, 137.90, 167.04, 170.36, 172.94; **LC-MS** (ESI) (90% H_2O to 100% MeCN in 10 min, then 100% MeCN to 15 min, DAD 220-600 nm), t_R = 4.61 min, 99% purity, *m*/*z* $[M + H]^+$ calcd for $C_{16}H_{13}N_2O_3$, 281.09; found, 281.1.

3-Phenoxypiperidine-2,6-dione (F)

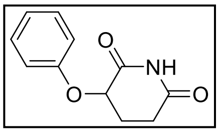

This compound was prepared by analogy with a previously reported method.[6] In brief, a stirred solution of phenol (0.94 mg, 10 mmol) in dry acetone (50 mL) were added 3-bromopiperidine-2,6-dione (2.30 g, 12 mmol), KI (0.17 g, 1 mmol) and K_2CO_3 (2.76 g, 20 mmol). The mixture was stirred at reflux for 16 h. It was then concentrated under reduced pressure, and the remaining residue was dissolved in H_2O (50 mL), and it was extracted with CH_2Cl_2 (3 × 25 mL). The combined organic layers were washed with H_2O (25 mL), dried over Na_2SO_4, filtered, and concentrated in vacuo. The crude product was dissolved H_2O (10 mL), washed with cyclohexane (10 mL), and the aqueous layer was placed in an Erlenmeyer flask for crystallization. After approximately 24 h, the precipitate was collected to obtain the title compound as a colorless solid. Yield: 32 mg (2%); mp 160-164 °C; R_f = 0.43 (20% EtOAc in petroleum ether); **^{1}H NMR** (500 MHz, DMSO-d_6) δ 2.16 – 2.06 (m, 1H), 2.24 – 2.16 (m, 1H), 2.63 – 2.55 (m, 1H), 2.76 – 2.66 (m, 1H), 5.19 (dd, J = 5.0, 10.8 Hz, 1H), 6.95 (t, J = 7.3 Hz, 1H), 7.00 (d, J = 8.1 Hz, 2H), 7.28 (t, J = 7.7 Hz, 2H), 10.89 (br s, 1H); **^{13}C NMR** (126 MHz, DMSO-d_6) δ 24.28, 29.85, 72.56, 115.67, 121.38, 129.49, 157.91, 171.51, 172.73; **LC-MS** (ESI) (90% H_2O to 100% MeCN in 10 min, then 100% MeCN to 15 min, DAD 220-600 nm), t_R = 3.88 min, 96% purity, m/z $[M + H]^+$ calcd for $C_{11}H_{12}NO_3$, 206.08; found, 206.0.

3-(Phenylamino)piperidine-2,6-dione(G)

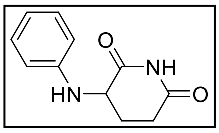

This compound was synthesized following a literature procedure.[7] In brief, to a solution of aniline (0.4 mL, 4.4 mmol) in DMF (20 mL) was added 3-bromopiperidine-2,6-dione (1.70 g, 8.8 mmol), and $NaHCO_3$ (7.43 g, 88 mmol). The reaction was stirred at 85 °C for 12 h. After cooling, the reaction mixture was quenched with ice water (70 mL), extracted with ethyl acetate (50 × 3 mL), washed with water (30 mL), brine (2 × 50 mL), dried over Na_2SO_4, and concentrated *in vacuo*. The crude product was purified by recrystallization from EtOAc to get an orange powder. Yield: 0.26 g (29%); mp 186-188 °C; R_f = 0.55 (50% EtOAc in petroleum ether); **^{1}H NMR** (600 MHz, DMSO-d_6) δ 1.91 – 1.81 (m, 1H), 2.14 – 2.07 (m, 1H), 2.61 – 2.54 (m, 1H), 2.78 – 2.69 (m, 1H), 4.33 – 4.27 (m, 1H), 5.77 (d, J = 7.5 Hz, 6.56 (t, J = 7.2 Hz, 1H), 1H), 6.67 (d, J = 7.9 Hz, 2H), 7.07 (t, J = 7.7 Hz, 2H), 10.76 (br s, 1H); **^{13}C NMR** (151 MHz, DMSO-d_6) δ 24.83, 30.89, 52.56, 112.78, 116.46, 128.95, 147.98, 173.24, 173.78; **LC-MS** (ESI) (90% H_2O to 100% MeCN in 10 min, then 100% MeCN to 15 min, DAD 220-600 nm), t_R = 3.40 min, 95% purity, m/z $[M + H]^+$ calcd for $C_{11}H_{13}N_2O_2$, 205.10; found, 205.1.

Selected NMR Spectra

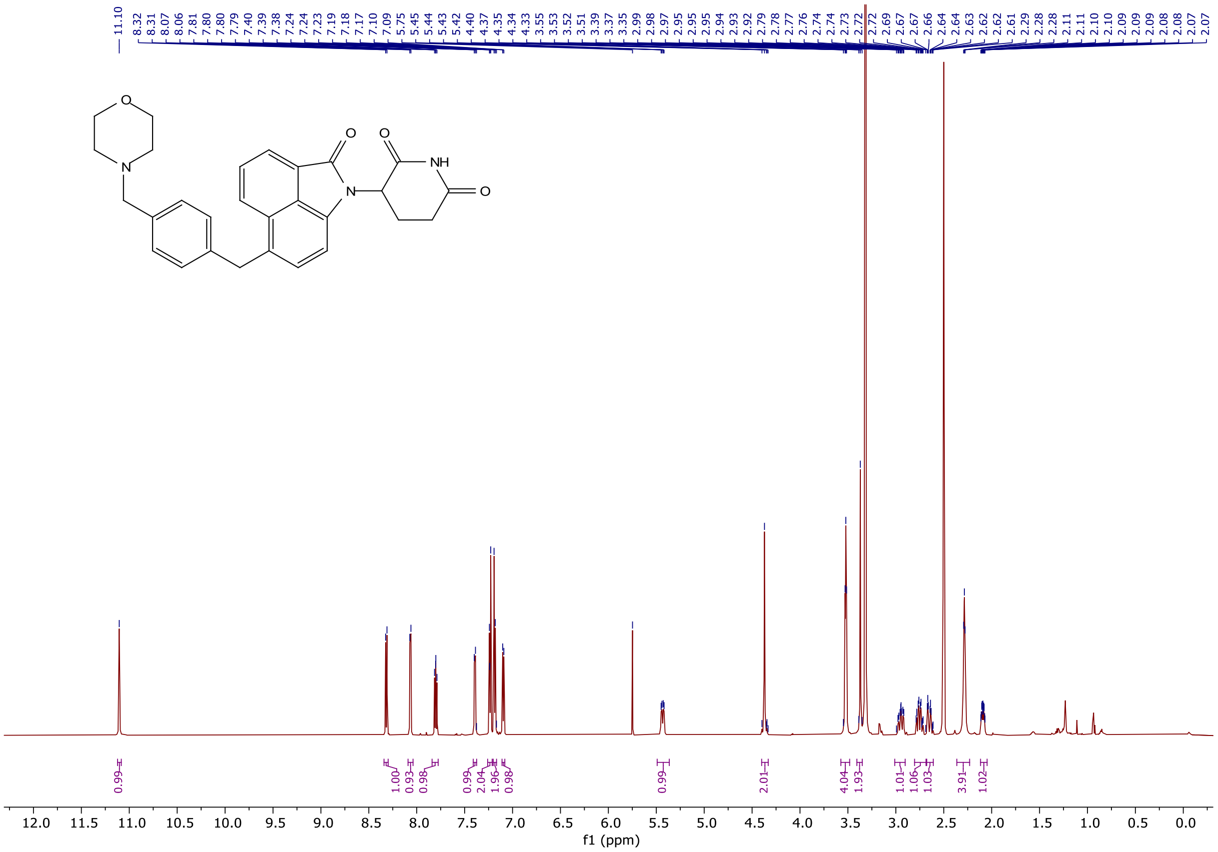

^{1}H NMR spectrum of **4**.

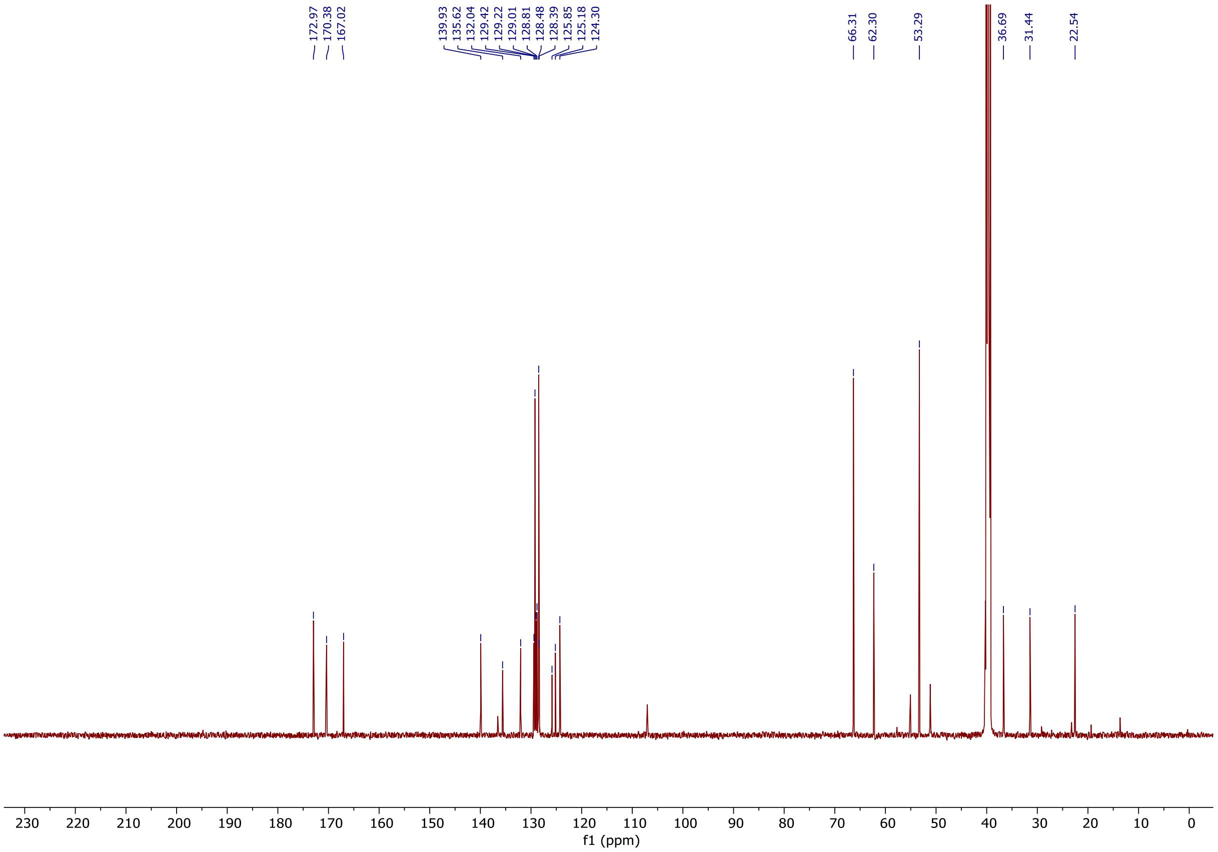

^{13}C NMR spectrum of **4**.

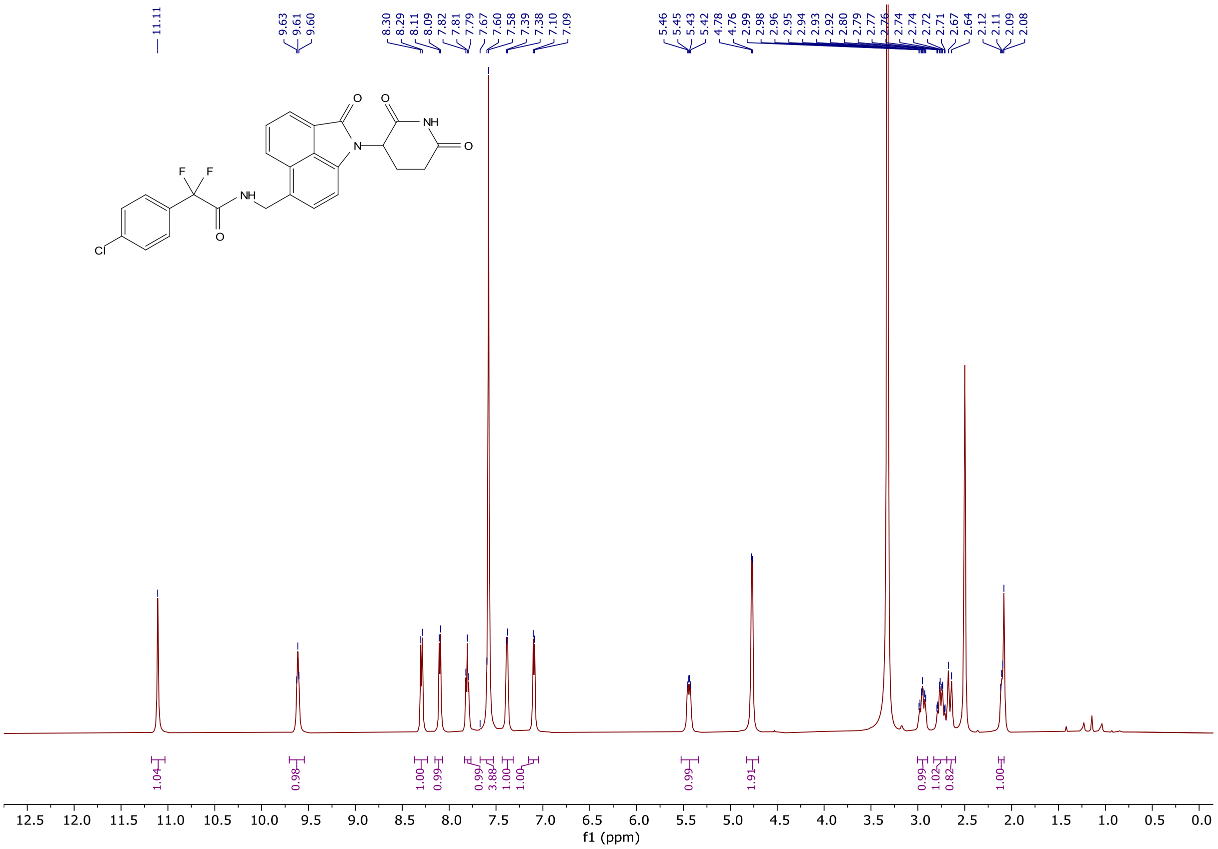

^{1}H NMR spectrum of **16**.

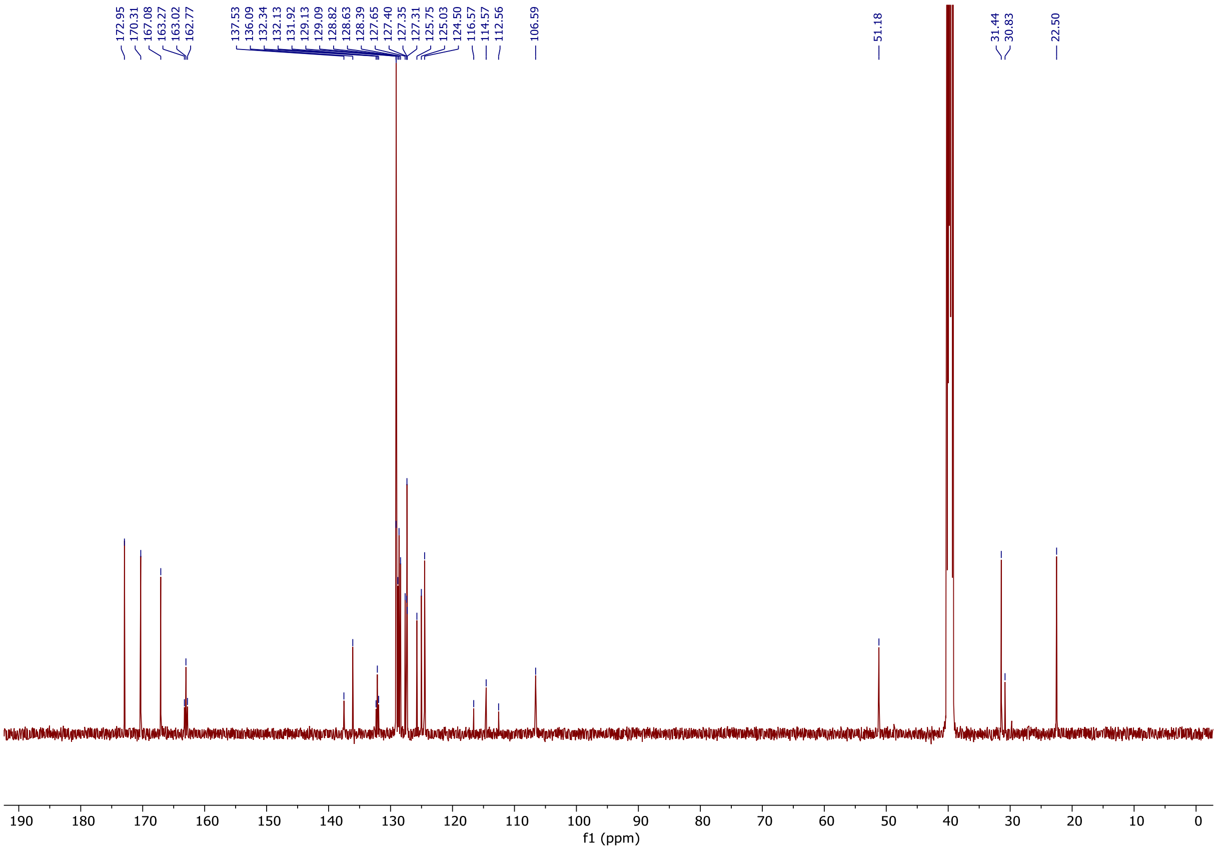

^{13}C NMR spectrum of **16.**

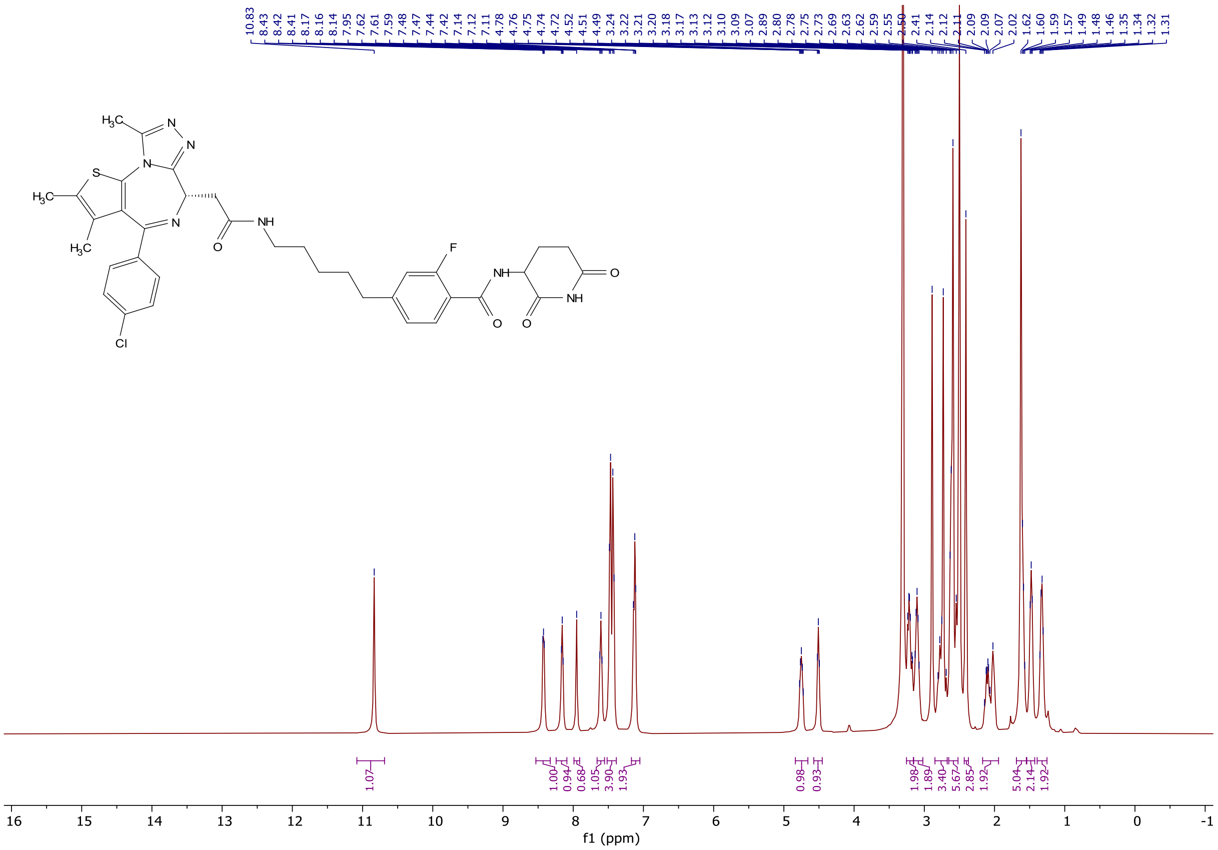

^{1}H NMR spectrum of **17**.

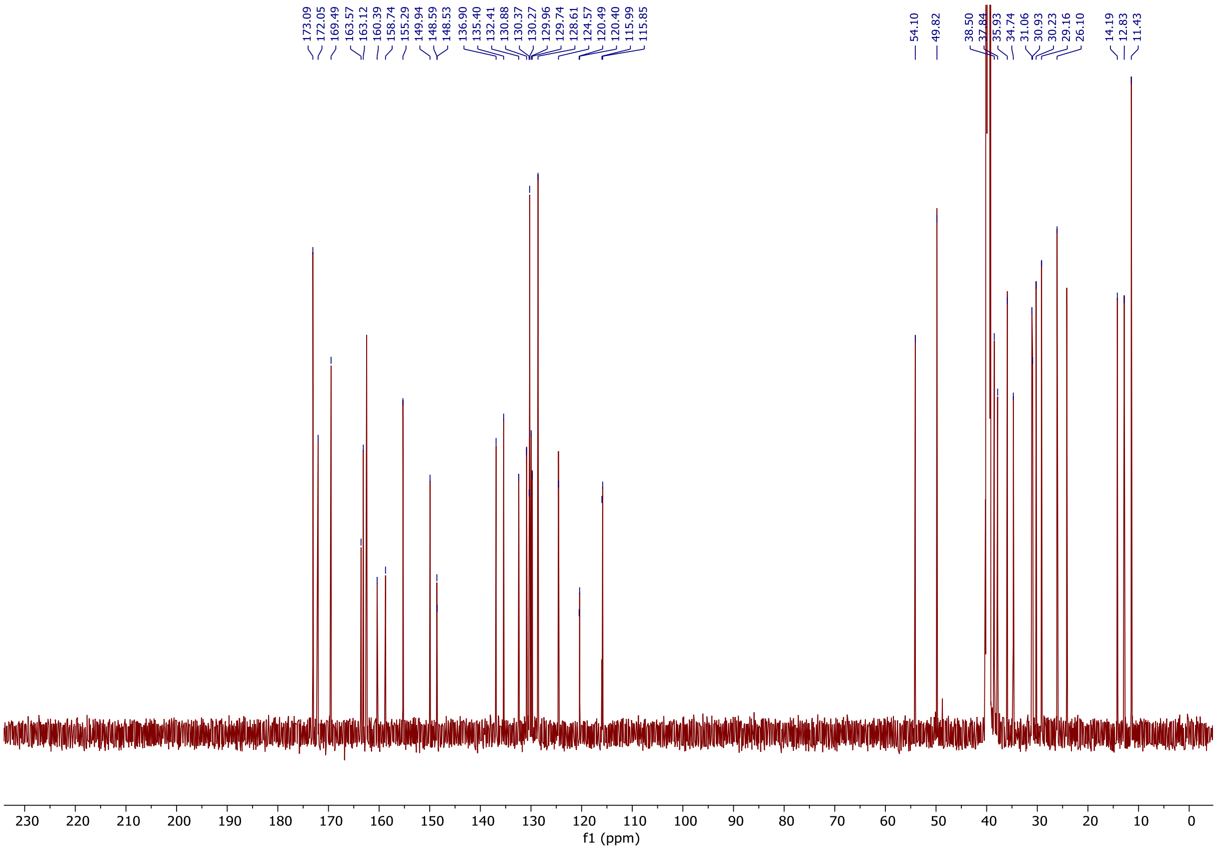

^{13}C NMR spectrum of **17.**

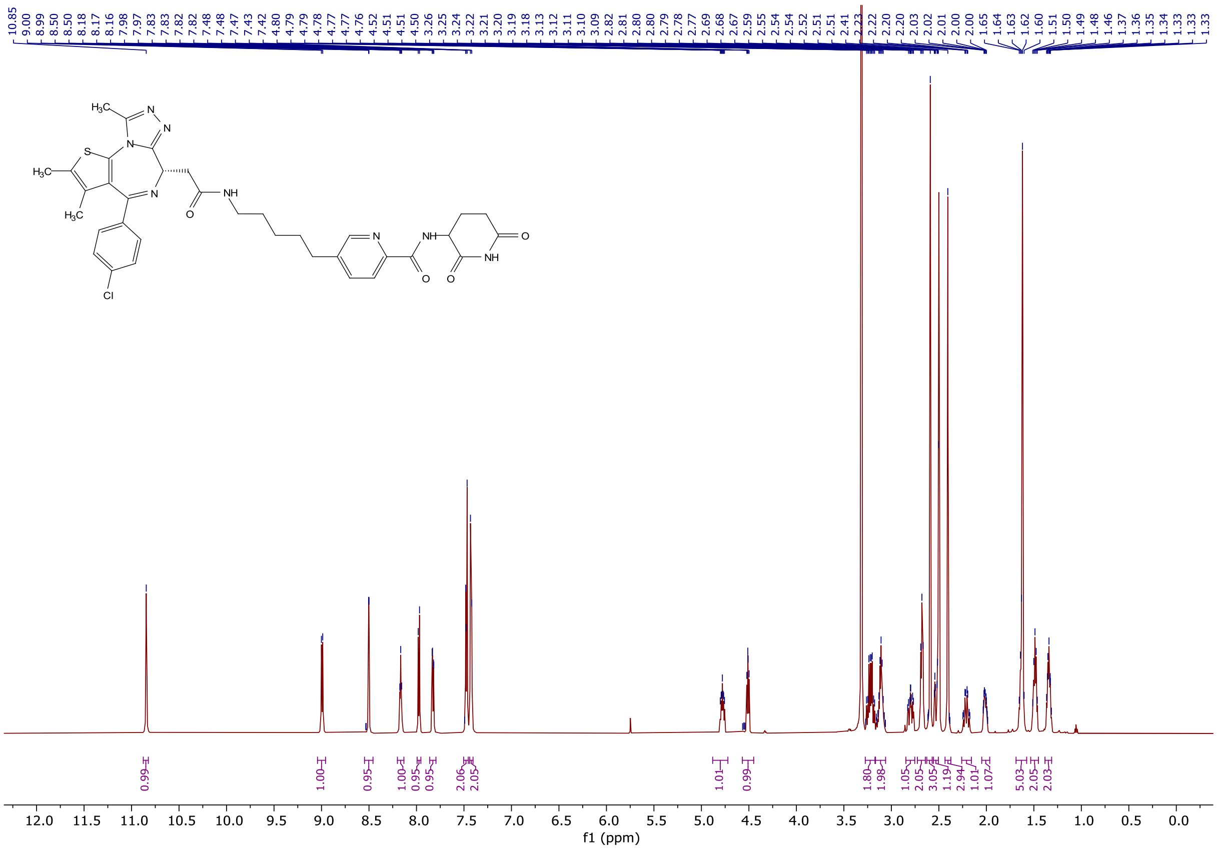

^{1}H NMR spectrum of **18**.

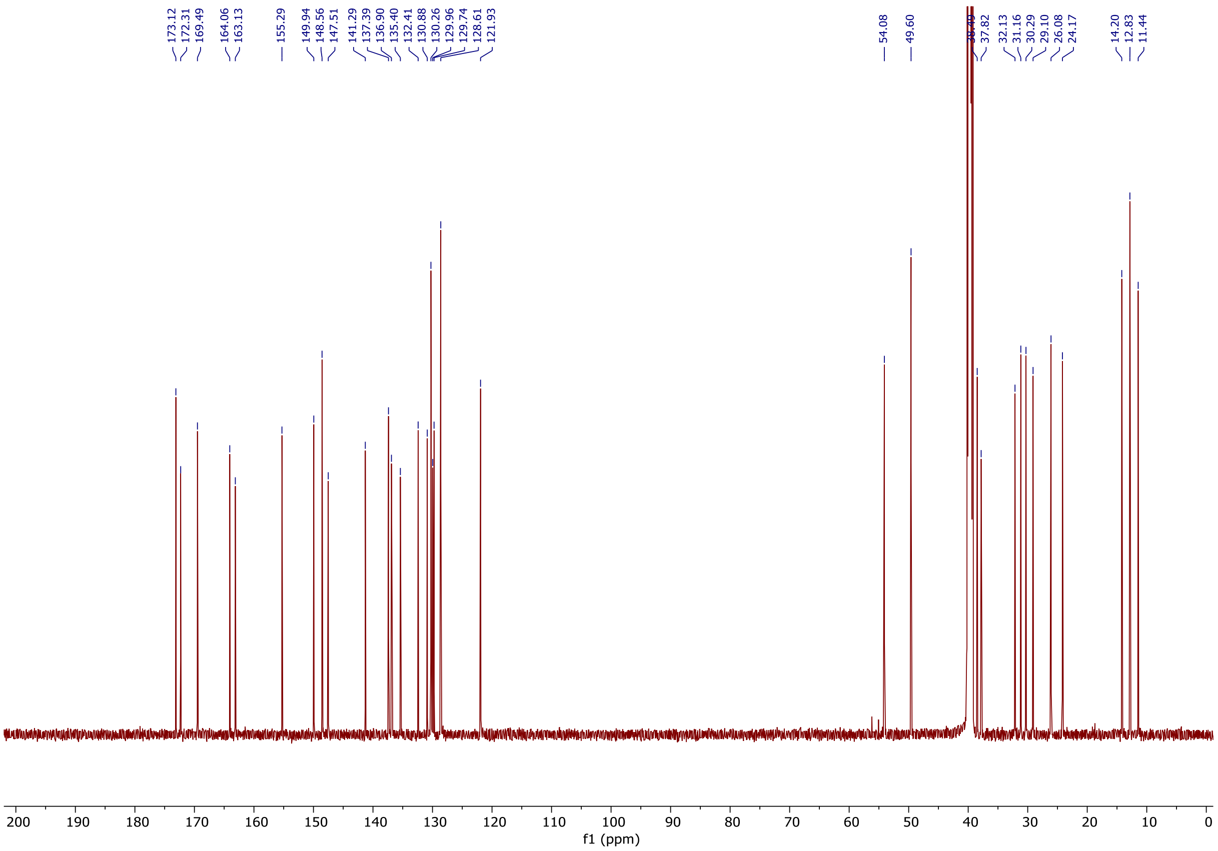

^{13}C NMR spectrum of **18.**

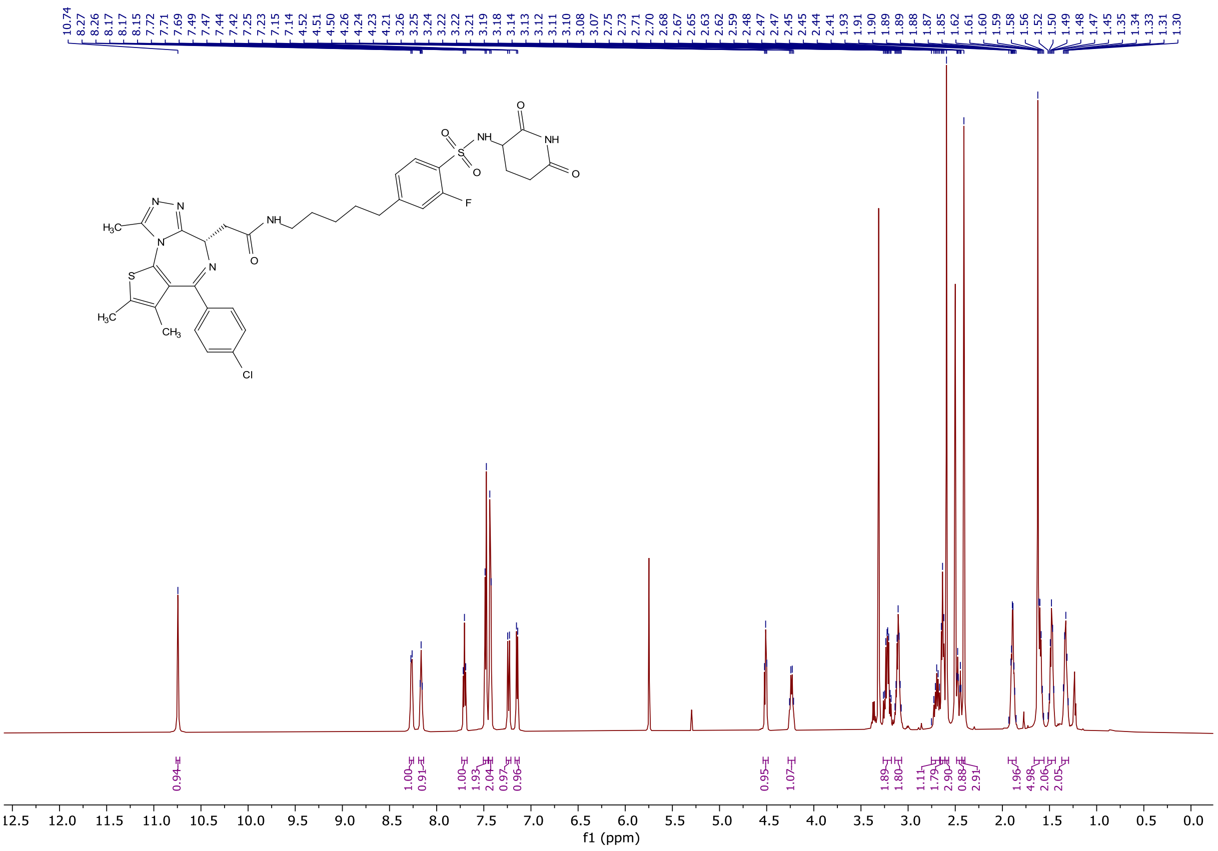

^{1}H NMR spectrum of **19**.

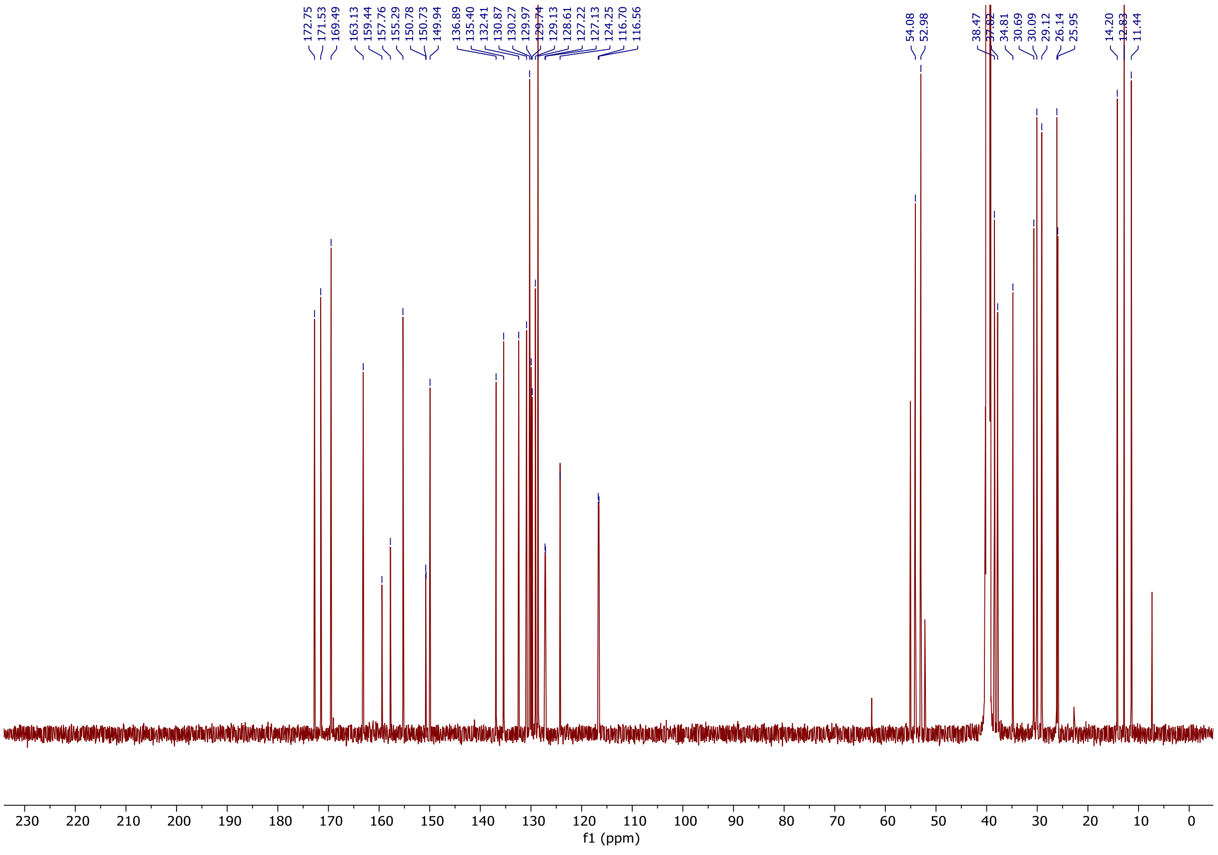

^{13}C NMR spectrum of **19.**

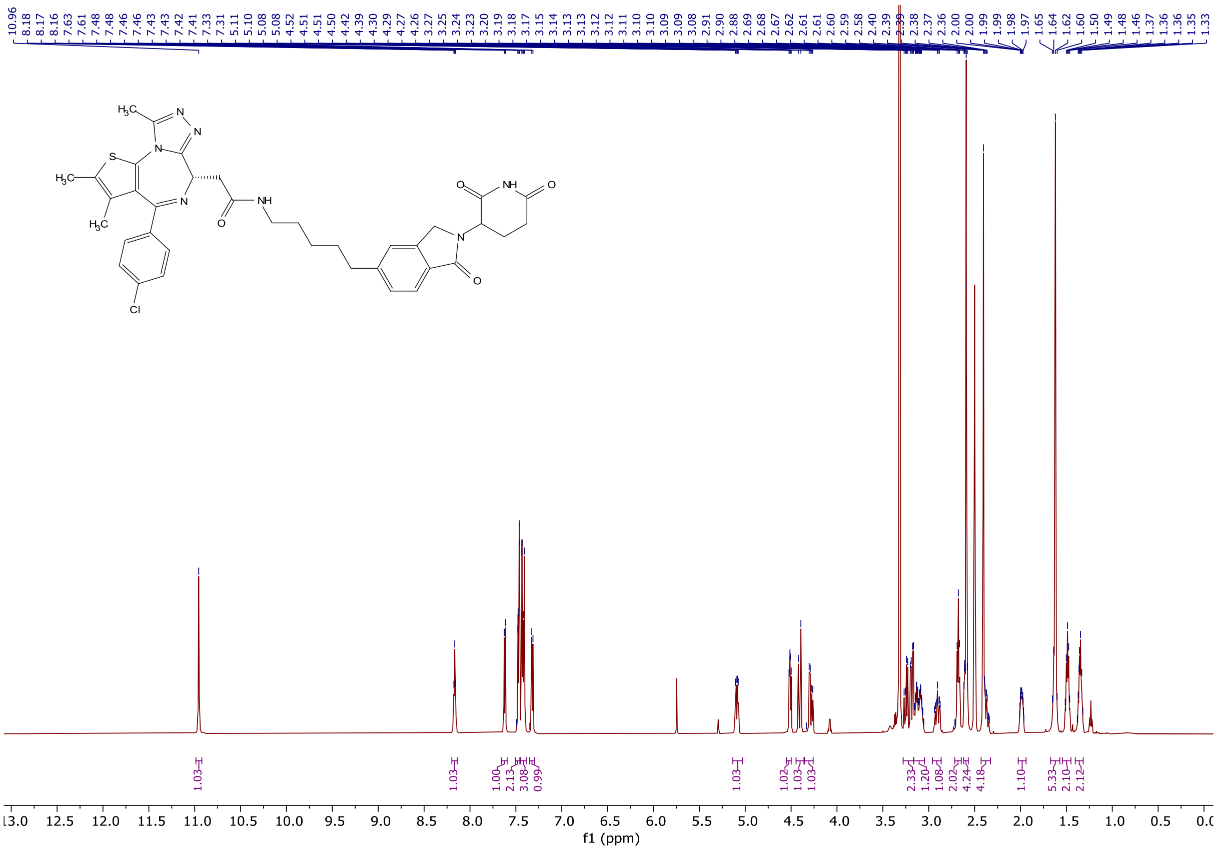

^{1}H NMR spectrum of **20**.

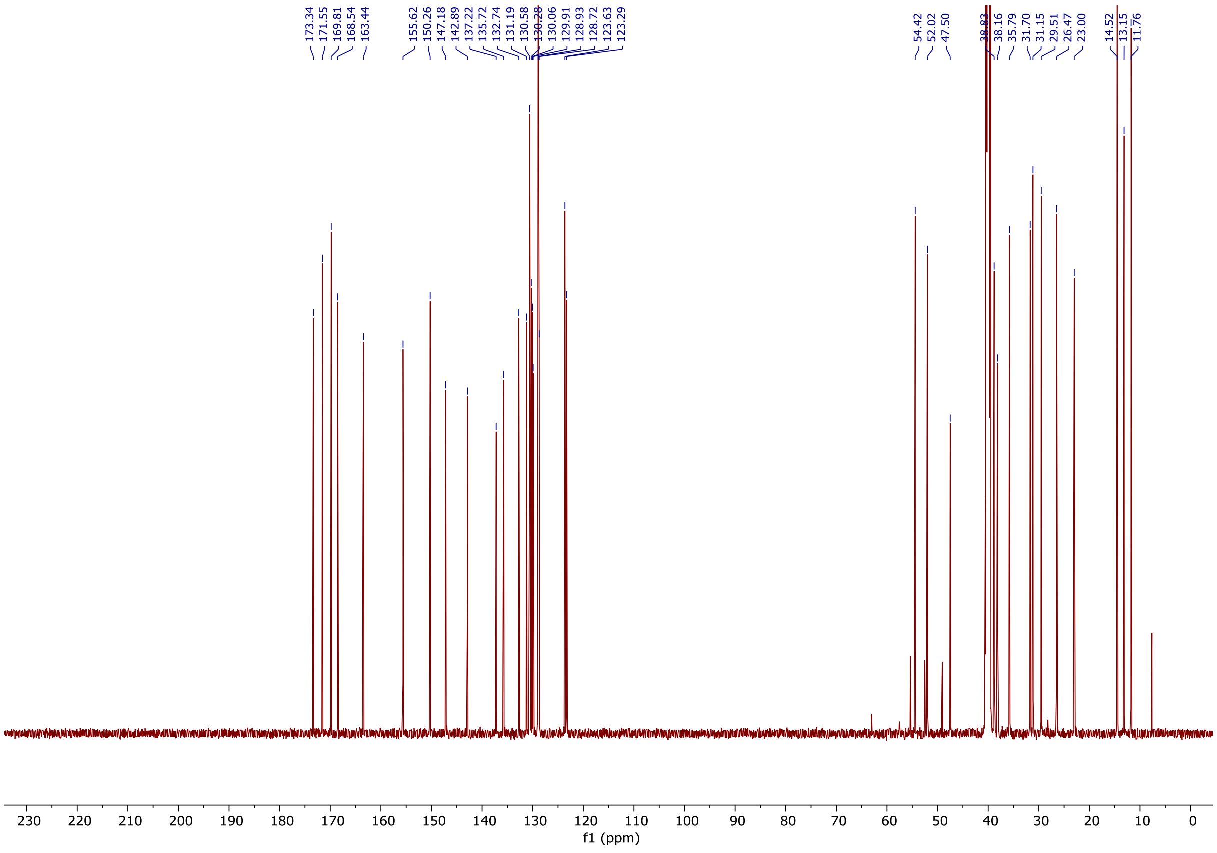

^{13}C NMR spectrum of **20.**

References

(1) Proj, M.; Knez, D.; Sosič, I.; Gobec, S. Redox Active or Thiol Reactive? Optimization of Rapid Screens to Identify Less Evident Nuisance Compounds. *Drug Discov. Today* **2022**, *27* (6), 1733–1742. https://doi.org/10.1016/j.drudis.2022.03.008.

(2) Steinebach, C.; Bricelj, A.; Murgai, A.; Sosič, I.; Bischof, L.; Ng, Y. L. D.; Heim, C.; Maiwald, S.; Proj, M.; Voget, R.; Feller, F.; Košmrlj, J.; Sapozhnikova, V.; Schmidt, A.; Zuleeg, M. R.; Lemnitzer, P.; Mertins, P.; Hansen, F. K.; Gütschow, M.; Krönke, J.; Hartmann, M. D. Leveraging Ligand Affinity and Properties: Discovery of Novel Benzamide-Type Cereblon Binders for the Design of PROTACs. *J. Med. Chem.* **2023**, *66* (21), 14513–14543. https://doi.org/10.1021/acs.jmedchem.3c00851.

(3) Min, J.; Mayasundari, A.; Keramatnia, F.; Jonchere, B.; Yang, S. W.; Jarusiewicz, J.; Actis, M.; Das, S.; Young, B.; Slavish, J.; Yang, L.; Li, Y.; Fu, X.; Garrett, S. H.; Yun, M.; Li, Z.; Nithianantham, S.; Chai, S.; Chen, T.; Shelat, A.; Lee, R. E.; Nishiguchi, G.; White, S. W.; Roussel, M. F.; Potts, P. R.; Fischer, M.; Rankovic, Z. Phenyl-Glutarimides: Alternative Cereblon Binders for the Design of PROTACs. *Angew. Chem. Int. Ed.* **2021**, *60* (51), 26663–26670. https://doi.org/10.1002/anie.202108848.

(4) Rathje, O. H.; Perryman, L.; Payne, R. J.; Hamprecht, D. W. PROTACs Targeting MLKL Protect Cells from Necroptosis. *J. Med. Chem.* **2023**, *66* (16), 11216–11236. https://doi.org/10.1021/acs.jmedchem.3c00665.

(5) Teng, M.; Lu, W.; Donovan, K. A.; Sun, J.; Krupnick, N. M.; Nowak, R. P.; Li, Y.-D.; Sperling, A. S.; Zhang, T.; Ebert, B. L.; Fischer, E. S.; Gray, N. S. Development of PDE6D and CK1α Degraders through Chemical Derivatization of FPFT-2216. *J. Med. Chem.* **2022**, *65* (1), 747–756. https://doi.org/10.1021/acs.jmedchem.1c01832.

(6) Yu, J.; Zhang, P.; Wu, J.; Shang, Z. Metal-Free C–N Bond-Forming Reaction: Straightforward Synthesis of Anilines, through Cleavage of Aryl C–O Bond and Amide C–N Bond. *Tetrahedron Lett.* **2013**, *54* (24), 3167–3170. https://doi.org/10.1016/j.tetlet.2013.04.028.

(7) Yu, J.; Zhang, P.; Wu, J.; Shang, Z. Metal-Free C–N Bond-Forming Reaction: Straightforward Synthesis of Anilines, through Cleavage of Aryl C–O Bond and Amide C–N Bond. *Tetrahedron Lett.* **2013**, *54* (24), 3167–3170. https://doi.org/10.1016/j.tetlet.2013.04.028.
